# CryoEM reveals that ribosomes in microsporidian spores are locked in a dimeric hibernating state

**DOI:** 10.1038/s41564-023-01469-w

**Published:** 2023-09-14

**Authors:** Mathew McLaren, Rebecca Conners, Michail N. Isupov, Patricia Gil-Díez, Lavinia Gambelli, Vicki A. M. Gold, Andreas Walter, Sean R. Connell, Bryony Williams, Bertram Daum

**Affiliations:** 1 Living Systems Institute, University of Exeter, Exeter, UK; 2 Department of Biosciences, Faculty of Health and Life Sciences, University of Exeter, Exeter, UK; 3 Center of Optical Technologies, Aalen University, Aalen, Germany; 4 Structural Biology of Cellular Machines, IIS Biobizkaia, Barakaldo, Spain; 5 Present Address: Crop Science Centre, Cambridge University, Cambridge, UK; 6 Present Address: MRC Laboratory of Molecular Biology, Cambridge Biomedical Campus, Cambridge, UK

**Keywords:** Immunology, Microbiology, Mechanisms of disease, Cryoelectron tomography, Cryoelectron microscopy

## Abstract

Translational control is an essential process for the cell to adapt to varying physiological or environmental conditions. To survive adverse conditions such as low nutrient levels, translation can be shut down almost entirely by inhibiting ribosomal function. Here we investigated eukaryotic hibernating ribosomes from the microsporidian parasite *Spraguea lophii* in situ by a combination of electron cryo-tomography and single-particle electron cryo-microscopy. We show that microsporidian spores contain hibernating ribosomes that are locked in a dimeric (100S) state, which is formed by a unique dimerization mechanism involving the beak region. The ribosomes within the dimer are fully assembled, suggesting that they are ready to be activated once the host cell is invaded. This study provides structural evidence for dimerization acting as a mechanism for ribosomal hibernation in microsporidia, and therefore demonstrates that eukaryotes utilize this mechanism in translational control.

## Abstract (web summary)

Electron cryo-microscopy revealed that ribosomes from microsporidian parasites were in a hibernating state, whereby the ribosomes form inactive pairs.

## Main

The ribosome is the central protein production hub of the cell and conserved throughout evolution. The process of protein translation is very energy expensive and can consume up to 40% of all cellular energy^1^. Under adverse environmental or physiological conditions, a large proportion of the cell’s adenosine triphosphate consumption can be saved by downregulating translation at the level of the ribosome^2^. This is achieved by various ribosomal inhibitors, a subset of which promote ribosomal hibernation^3^. These so-called hibernation factors inactivate ribosomes by two mechanisms: either by locking individual ribosomes in a state that is incompatible with translation or through promoting the formation of dimeric, 100S ribosomes. Either mechanism prevents the dissociation of the small and large subunit, which is an essential prerequisite for messenger RNA loading and translation^1^. Moreover, ribosome dimerization protects the ribosomes from degradation by RNases^4^.

The mechanisms of ribosomal hibernation are particularly well investigated in bacteria^4^. In stationary growth phases or under conditions of nutrient-starvation, two forms of hibernating ribosomes appear to co-exist. In *Escherichia coli* these are a 70S ribosome, inactivated by the hibernation factor RaiA, and a 100S ribosome dimer, stabilized by the binding of up to two hibernation factors called ribosome modulation factor (RMF) and hibernation promoting factor (HPF)^2,5,6^. In combination, these factors block ribosome function by binding and occluding the decoding centre, the mRNA-binding channel, and the acceptor (A) and peptidyl (P) transfer RNA sites^6–8^. A recent cryo-electron microscopy (cryoEM) study of hibernating ribosomes from *E.* *coli* revealed a 100S particle that, in addition to HPF and RMF, was also bound to the factor bS1, as well as a deacetylated tRNA in the exit (E) site. Here, bS1 is stabilized by RMF and together both factors sequester the anti-Shine-Dalgarno sequence of the 16S ribosomal RNA. In addition, the E-tRNA is stabilized by HPF, which itself occludes the binding site for the mRNA as well as A- and P-site tRNAs^9^. Many other bacteria, such as *Thermus thermophilus*, do not express RMF and instead rely on an extended version of HPF, HPF_long_, for 100S ribosome formation^4^.

In eukaryotes, ribosomal hibernation has so far been structurally characterized at the single (80S) ribosome level. In hibernating yeast ribosomes, ribosomal function is blocked by the protein Lso2, which sequesters the mRNA-binding pocket as well as the polypeptide exit tunnel concomitantly^10^. In mammals, various types of hibernating ribosomes can be found. In hibernating ribosomes isolated from human cell culture, two silencing states appear to co-exist: a non-rotated state bound to the Lso2 homologue CCDC124, as well as EBP1 at the polypeptide exit site and a second rotated state, where CCDC124 is exchanged by SERBP1 and eEF2 (ref. ^10^). Whereas CCDC124/SERBP1 and eEF2 are analogous in occupying the mRNA entry channel and blocking the A and P sites, EBP1 prevents the stalled ribosome’s futile interaction with proteins interacting with the nascent polypeptide chain, including the ribosome associated complex, signal recognition particle, secretory protein 61 and *N*-α-acetyltransferase A (ref. ^10^).

An alternative mammalian hibernation mechanism has been discovered in ribosomes isolated from reticulocytes. Here, 80S ribosomes are inactivated by binding interferon-related developmental regulator 2. Interferon-related developmental regulator 2 occupies the P and E sites and sequesters the mRNA with its alpha-helical C-terminus. At the same time, a deacylated tRNA is bound to a non-canonical site beyond the E site, called the Z site. It was hypothesized that stably bound deacetylated Z-tRNA may present a hallmark of stalled ribosomes under scenarios of amino acid depletion, or where translational factors are limiting^11^. Biochemical evidence and negative stain electron microscopy suggest that ribosomal dimerization also occurs in rat cells during amino acid starvation^12^. However, so far, no structure of a eukaryotic ribosome dimer is available.

By combining cryo-electron tomography (cryoET) with subtomogram averaging and single-particle cryoEM, we investigated the structure of eukaryotic hibernating ribosome dimers in the microsporidian species *Spraguea lophii*. Microsporidia are single-celled eukaryotic intracellular parasites with species infecting almost all animal lineages^13^. They have the potential to cause debilitating disease in immuno-compromised humans and severe deleterious impacts on food production^14^. Microsporidia begin their life cycle as dormant spores that need to enter and exploit the energy metabolism of a host cell to proliferate^13^. They achieve entry into host cells via a pre-formed, tightly coiled ‘polar tube’ (PT) that is rapidly expelled from the dormant spore on germination^15^. The PT then penetrates the host-cell membrane, and the spore content (sporoplasm) is swiftly transported down the tube into the host cell^15^. As the PT is usually not more than 150 nm wide, this presents a perfect opportunity to image hibernating ribosomes by cryoET in situ.

Single-particle cryoEM of ribosomes isolated from the microsporidian species *Varimorpha necatrix*^16^, *Paranosema locustae*^17^ and *Encephalitozoon cuniculi*^18^ have revealed structures of monomeric ribosomes in a hibernating state. These studies confirm the highly reduced nature of microsporidian ribosomes that show a drastic loss of the expansion segments characteristic of eukaryotic rRNA, but that have largely retained the core set of typical ribosomal proteins. The complexes are similar in size to their bacterial counterparts and are thus designated as 70S ribosomes^16,17,19^. Similar to yeast, the ribosomes of *P.* *locustae* were shown to be inhibited by the protein Lso2, which spans the mRNA decoding site and the large subunit (LSU) tRNA binding site. In contrast, hibernating ribosomes of *V.* *necatrix* were bound to two inhibiting factors, called MDF1 (a conserved eukaryotic protein) and MDF2 (a protein only identified in *V.* *necatrix, N.* *ceranae* and *N.* *apis*). While MDF1 binds the E site of the SSU and stabilizes the ribosome in a conformation incompatible with translation, MDF2 blocks the P site as well as the polypeptide exit tunnel of the ribosome^16^. In *E.* *cuniculi*, the ribosome was bound to MDF1 in the E site, acting as a mimic of a deacylated tRNA molecule^18^.

By investigating PTs ejected from the species *S.* *lophii*, we find that sporoplasm traversing the PT is occasionally densely packed with hibernating 100S ribosome dimers, which so far have only been characterized in detail in bacteria. The architecture of the 100S ribosomes is markedly distinct from any of the bacterial structures known so far. In this article, through the study of this unique group of organisms, we present the first structural evidence of ribosomal dimerization as a mechanism for ribosomal hibernation in eukaryotes.

## *S. lophii* ribosomes form translationally dormant 100S dimers

**Fig. 1:**
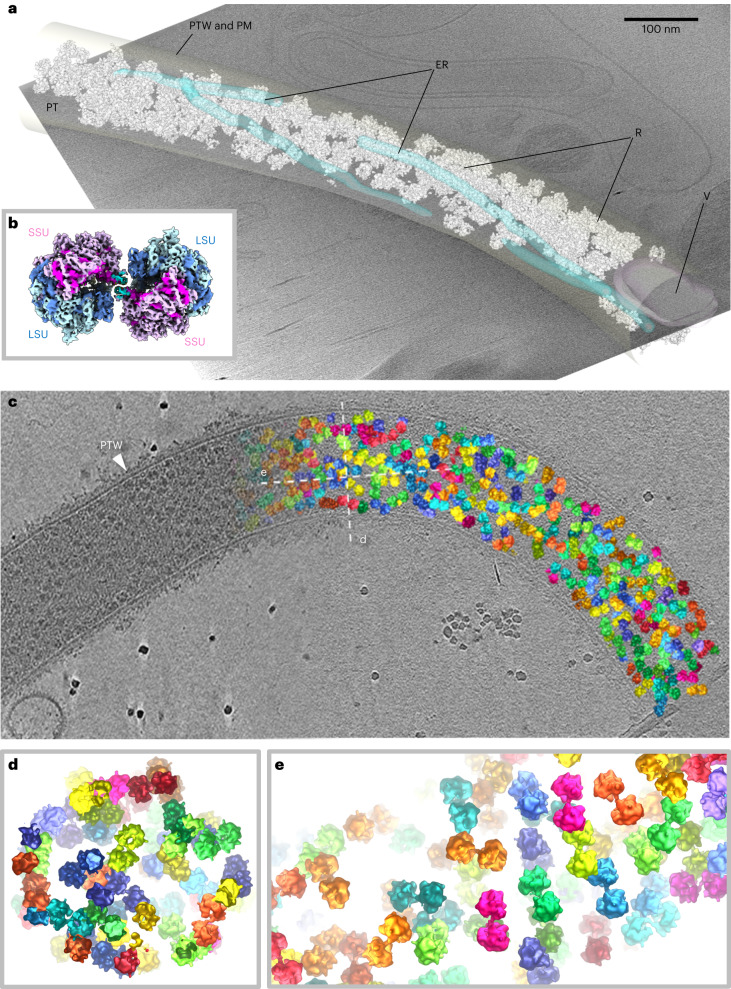
Structure and organization of the *S. lophii* ribosome dimer in situ. **a**, A segmented tomogram of a PT, showing ER (transparent blue), a vesicle (V, transparent magenta) and ribosomes (R, white). The PT wall (PTW) and plasma membrane (PM) are transparent grey. **b**, A subtomogram average of the *S.* *lophii* ribosome dimer (composite of two half-dimers) at 10.8 Å resolution, showing the SSU in shades of pink and the LSU in shades of blue. **c**, Organization of ribosome dimers inside a PT showing the original tomographic slice on the left and subtomogram averages of ribosome dimers placed back into the tomogram on the right (various colours). **d**,**e**, Cross-sections through the PT from areas indicated by the dotted lines (designated **d** and **e**) in **c**.

**Extended Data Fig. 1:**
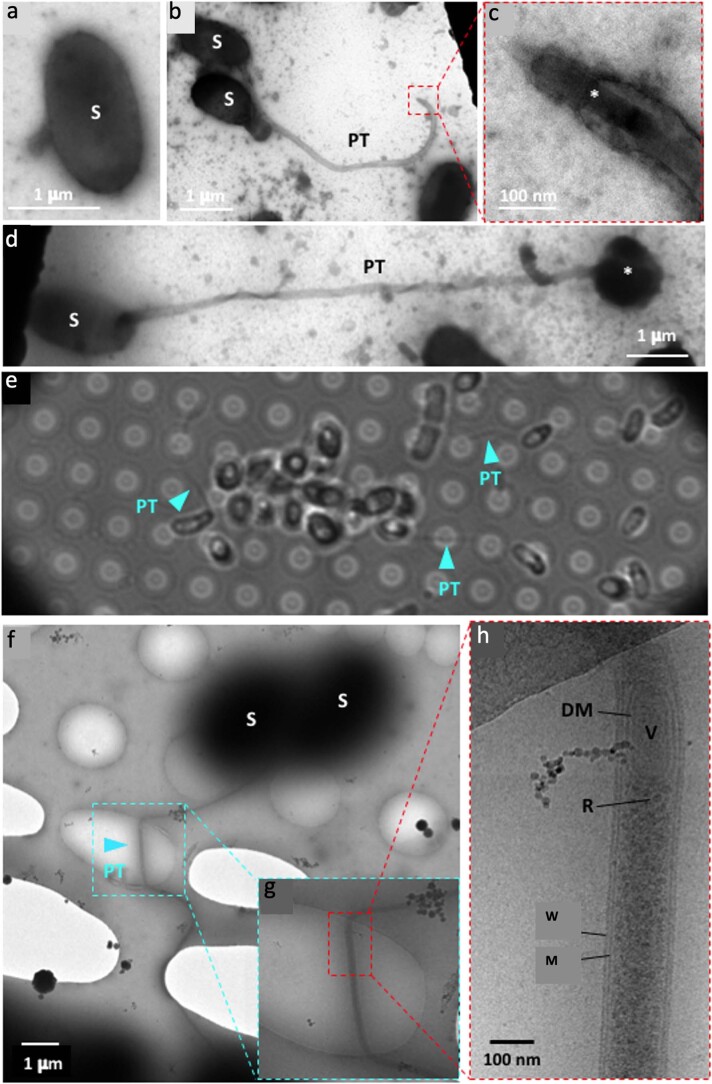
Electron microscopy of microsporidia germinated on EM grids. **a**–**d** Negative stain EM of **a** a dormant spore (S), **b** a germinated spore with polar tube (PT) extended, **c** the tip of a PT with sporoplasm (*) passing through at high magnification and **d** a germinated spore post transfer, with sporoplasm egressing terminally (*). **e** Bright field light microscopy showing that microsporidia can be germinated on holy carbon (Quantifoil) grids. PTs are indicated with blue arrowheads. **f-h** CryoEM of microsporidia germinated on Multi-A Quantifoil grids, showing a PT emerging from a spore at three different magnifications. The PTs span Quantifoil holes, which is an important requirement for cryoET. **g**, **h** Higher magnification images of dashed area in **f**. **h** The PT is confined by a membrane (M) and proteinaceous PT wall (W). Transported content is visible. (R) ribosomes, (V) vesicle and (DM) double membrane. These images are representative of hundreds of micrographs collected from multiple (5) sample preparations.

To investigate the structure of hibernating ribosomes in situ, we germinated microsporidian spores of the species *S.* *lophii* on the cryoEM grid and plunge froze these grids in liquid ethane. Examination of the samples in the electron microscope showed spores that were too dense to be penetrated by the electron beam. Many of these spores exhibited up to 50-µm-long and 150–200-nm-wide extensions, which were identified as PTs (Extended Data Fig. 1). We recorded cryoEM projections, as well as tomograms of PT segments and analysed the data in detail. Close inspection of the PTs revealed that they were confined by a fuzzy, probably proteinaceous coat, which was often lined with a membrane on the inside (Extended Data Fig. 1h and Supplementary Fig. 1). These membrane-lined PTs contained clearly recognizable cytosolic content, within which ribosomes could be distinguished (Fig. 1a,c, Extended Data Fig. 1h and Supplementary Fig. 1).

**Extended Data Fig. 2:**
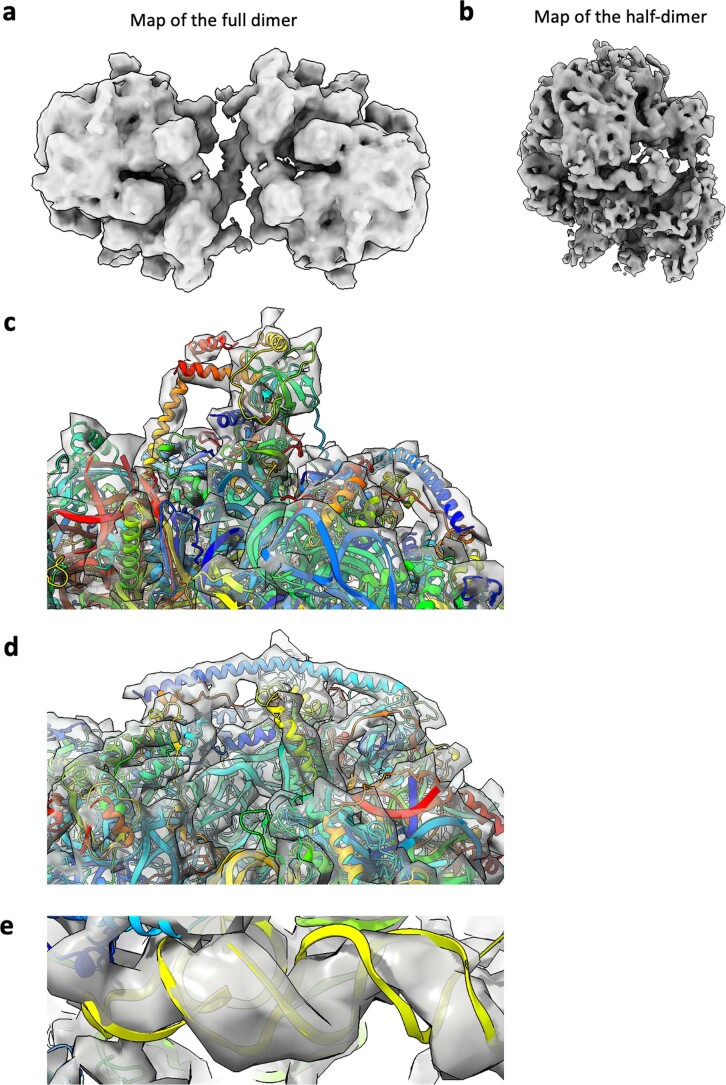
Quality of fit of atomic coordinates to subtomogram averaging maps. **a** Map of the dimer at 14.3 Å resolution. **b** Masked average of the half dimer at 10.8 Å resolution. **c-e** Three different regions of the ribosome showing the fit of the atomic coordinates obtained by single particle analysis to the half dimer map obtained by subtomogram averaging.

We manually selected 6,505 ribosomes within the PTs from *S.* *lophii* and subjected them to subtomogram averaging in Relion 3.1 (ref. ^20^) followed by M (ref. ^21^) (Supplementary Fig. 2). The resulting map (Fig. 1b and Extended Data Fig. 2) with an estimated resolution of 14.3 Å (as per Fourier Shell Correlation (FSC) 0.143; Supplementary Fig. 3) shows a dimeric complex of two 70S ribosomes interacting via the small subunit (SSU) in an anti-parallel head-to-head orientation. By focusing on one ribosome per dimer, the map was further refined to 10.8 Å resolution (as per FSC 0.143; Extended Data Fig. 2b and Supplementary Fig. 3; for 0.5 FSC and map/model FSC values, see Methods and Supplementary Fig. 3). Three-dimensional (3D) classification indicated that ~80% of the ribosome particles in the PT are 100S dimers, while ~20% contributed to a class accounting for monomeric (70S) ribosomes (Supplementary Fig. 4).

Placing the subtomogram average back into the original particle positions within the tomogram showed variable packing of ribosome dimers within the PT (Fig. 1c–e). While in some instances the ribosome dimers are almost at touching distance, other areas contain pockets of cytosol between them. A statistical analysis of distances and angular orientations between closest neighbours revealed no higher order of organization (Supplementary Fig. 5), indicating an absence of polysomes or any specific packing of the hibernating dimers. In addition, 3D classification also did not show any ribosomes associated with a membrane (Supplementary Fig. 4), even though structures reminiscent of endoplasmic reticulum (ER) tubules were present in some of the tomograms (Fig. 1a). These results suggest that the ribosomes in the PTs are not actively translating or engaged with a membrane translocase, such as the SEC translocon.

## Single-particle cryoEM of hibernating *S. lophii* ribosomes

To obtain a better understanding of the structure of the hibernating ribosome from *S.* *lophii*, we isolated ribosomes from spores using sucrose gradient centrifugation (Supplementary Fig. 6a), prepared cryoEM samples using ultrathin carbon-coated grids and recorded single-particle datasets. Two-dimensional classification showed that the particles were 70S ribosome monomers (Supplementary Fig. 6b), suggesting that the sample preparation procedure did not maintain the dimer contacts. This is in accordance with the single-particle structures of isolated hibernating ribosomes of the microsporidia *V.* *necatrix*, *P.* *locustae* and *E.* *cuniculi*, where dimers were also not observed.

**Fig. 2:**
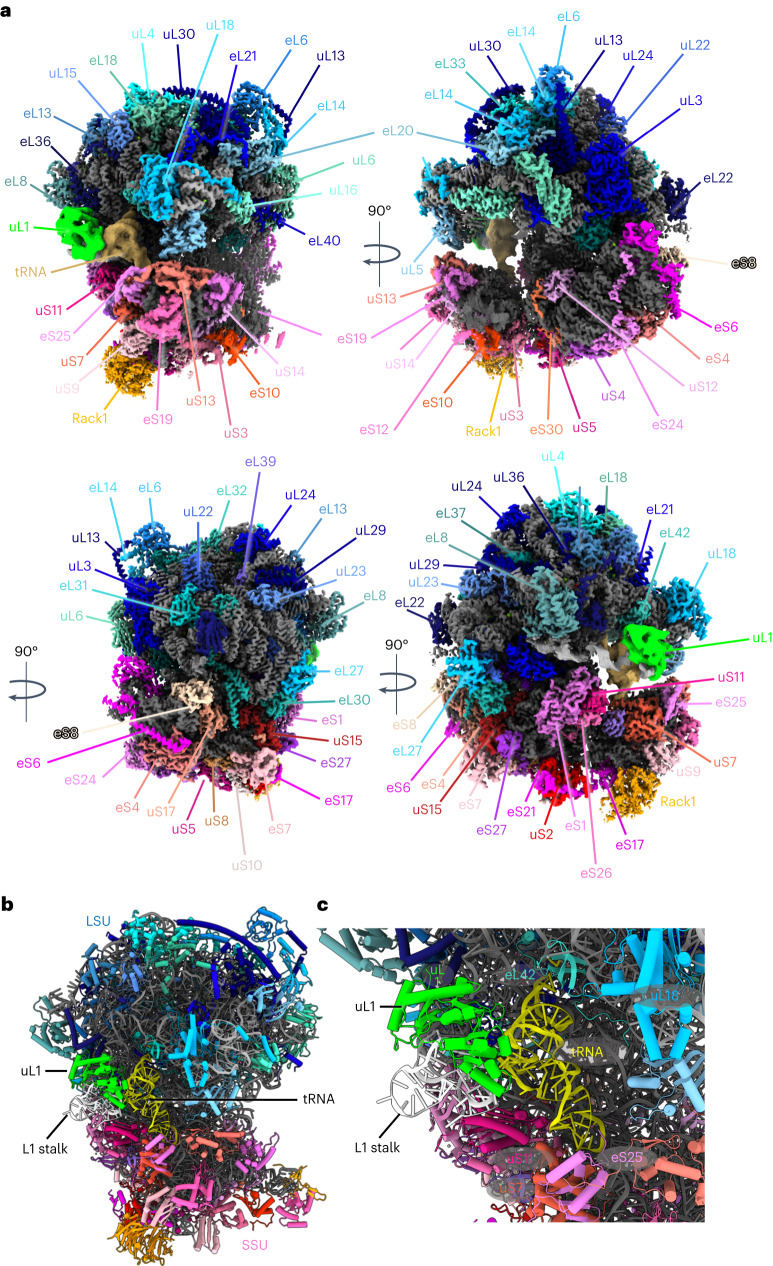
Single particle structure of the *S. lophii* 70S ribosome at 2.3–2.9 Å resolution. **a**, Various views of the ribosome, showing the protein chains of the LSU in shades of blue, the SSU in shades of red and the rRNA in grey. Subunit names are indicated. **b**, Atomic model of the ribosome with uL1 in lime green and tRNA in yellow. **c**, Magnified view of the E site of the ribosome showing deacetylated tRNA in yellow, L1 rRNA in white and protein uL1 in lime green. The tRNA interacts with protein uS7 of the SSU, proteins eL42 and uL1 of the LSU and rRNA.

**Extended Data Fig. 3:**
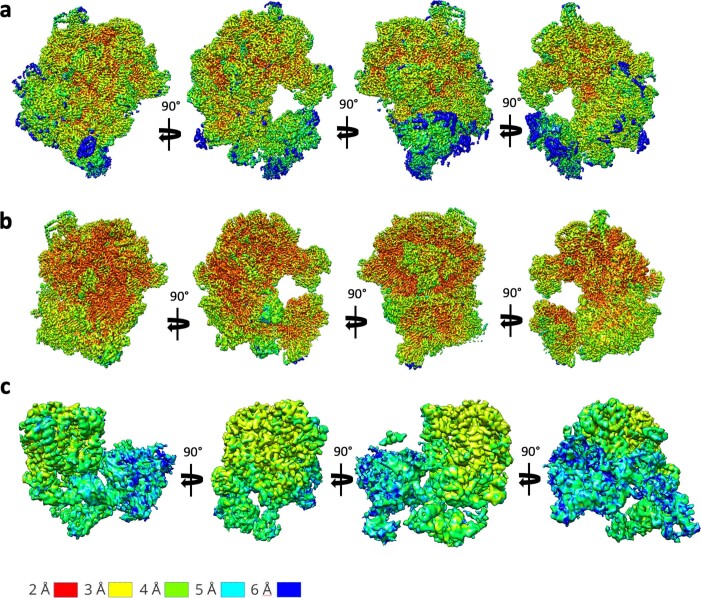
Local resolution maps of the single-particle analysis. **a** Whole-map refinement of the best 3D class, **b** 3-body refinement and **c** masked classification of the L1 stalk.

**Extended Data Fig. 4:**
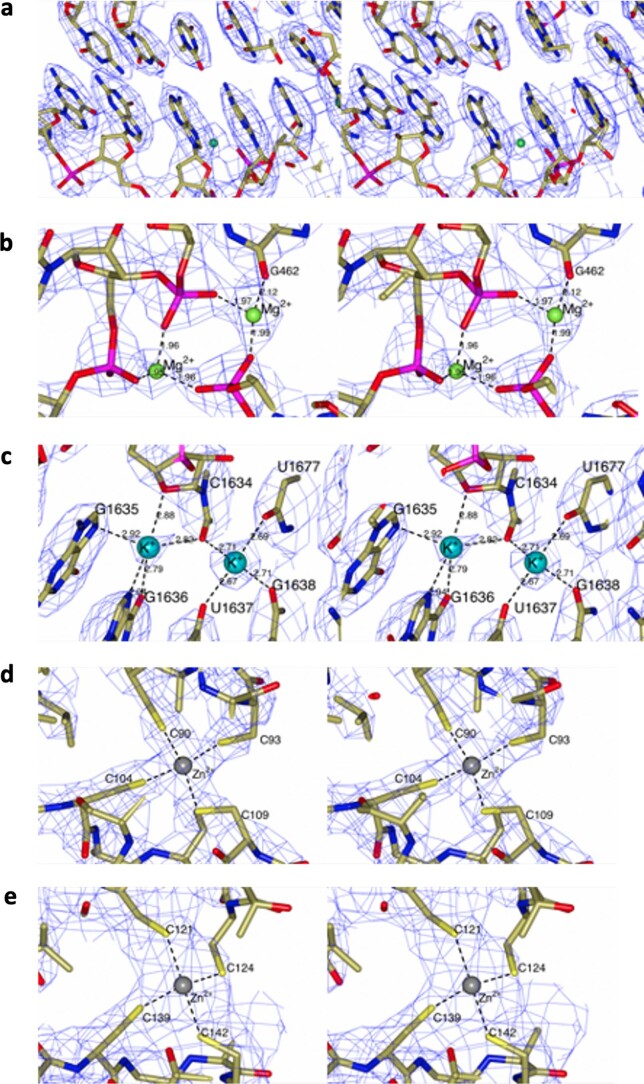
Representative close-ups of the cryoEM density. Stereograms showing sections of the cryo-EM density for regions of the whole-ribosome map (**a-d**) and SSU head map (**e**). **a** Representative section in the vicinity of G146 within the L50 rRNA clearly shows the difference between adenine and guanine bases. **b** Density peaks are modelled as Mg^2^^+^ ions on the basis of short (2.0 - 2.1 Å) coordinating distances to oxygen ligands. **c** K^+^ ions are assigned on the basis of longer coordinating distances (2.6 - 2.9 Å) which may include nitrogen ligands. **d-e** Zn^2+^ ions modelled in eL40 and eS31 chains.

Using multibody refinement in Relion 3.1, we were able to obtain a map of the *S.* *lophii* 70S ribosome at 2.26–2.79 Å resolution (Supplementary Figs. 7 and 8 and Extended Data Fig. 3), which exceeds the resolution previously achieved for microsporidian ribosomes^16–18^. On the basis of this map, we built an atomic model of the *S.* *lophii* 70S ribosome, consisting of 71 protein chains and 96.8% of the rRNA sequence (Fig. 2a, Supplementary Fig. 9 and Extended Data Fig. 4). Comparing our structure with previously published data showed that the 70S ribosome of *S.* *lophii* is in its non-rotated conformation (Supplementary Fig. 10), similar to structures of the ribosome of *P.* *locustae* but in contrast to those of *V.* *necatrix* and *E.* *cuniculi*. As seen in these other microsporidian structures, the *S.* *lophii* ribosome shows a drastic reduction in the expansion segments of the rRNAs relative to other eukaryotes. However, as in *P.* *locustae*, predicted 18S and 28S rRNAs are slightly longer than counterparts in *E.* *cuniculi* (68 and 129 nucleotides longer, respectively), indicating that rRNA reduction is slightly less pronounced in these earlier-branching lineages.

**Extended Data Fig. 5:**
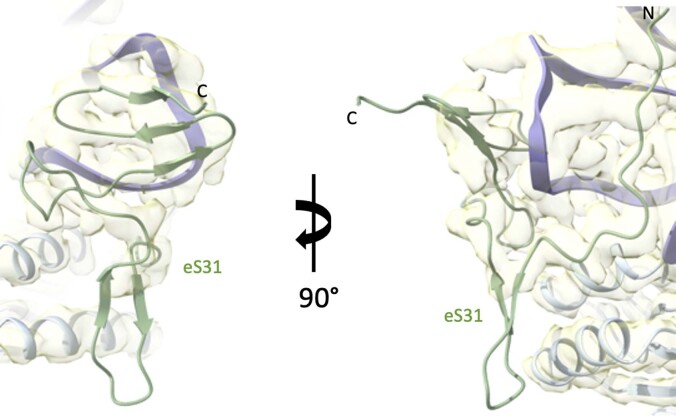
Atomic model of subunit eS31. The structure of *S. lophii* eS31 (green ribbon) was predicted in Alphafold2 and refined against the cryoEM map of the *S. lophii* ribosome (transparent yellow). The neighbouring 16S rRNA (purple ribbon) and the protein subunit eS12 (light grey ribbon) are also shown.

We did not observe a density for the large ribosomal protein subunit eL29, which was present in the previously published structures of microsporidian ribosomes from *V.* *necatrix*, *P.* *locustae* and *E.* *cuniculi*. In accordance with this, we find that the corresponding gene is absent from the *S.* *lophii* genome, suggesting a loss within this microsporidian lineage. However, our map does contain a density for the C-terminal domain of the SSU protein eS31 (one of two ribosomal ubiquitin fusion proteins; structure predicted by Alphafold2; Extended Data Fig. 5). This subunit was also observed in the *V.* *necatrix and E.* *cuniculi* ribosomes but is missing from the *P.* *locustae* structure.

**Extended Data Fig. 6:**
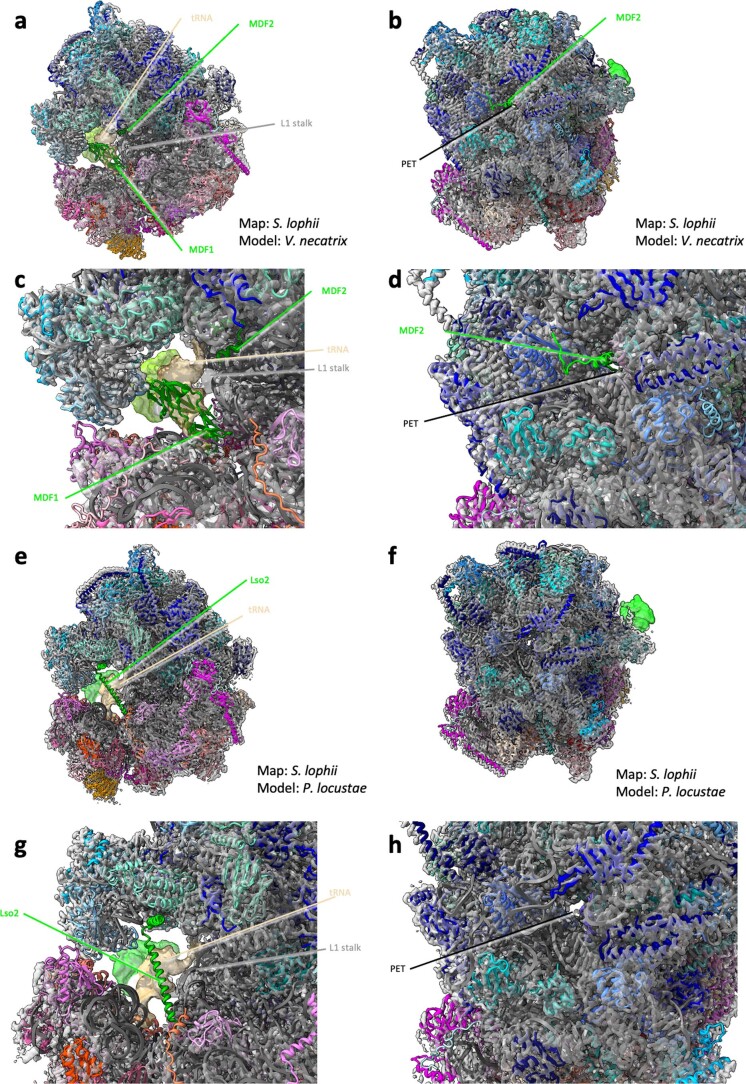
Absence of hibernation factors in the *S. lophii* single particle map. The single particle map of the *S. lophii* ribosome shows no densities for hibernation factors found in the microsporidian species *V. necatrix* and *P. locustae*. **a, b** Atomic model of the *V. necatrix* ribosome (multicolour) fitted into the density map of *S. lophii* (transparent grey; L1 stalk density shown as green surface and tRNA density shown as beige surface). The hibernation factor MDF1 blocking the mRNA and tRNA binding sites (green ribbon) is shown in **a** and the hibernation factor MDF2 occupying the polypeptide exit tunnel (PET) is shown with the same colour in **b**. **c, d** Close-up views of hibernation factors MDF1 and MDF2 from the *V. necatrix* structure. **e, f** Atomic model of the *P. locustae* ribosome (multicolour) fitted into the density map of *S. lophii* (transparent grey; L1 stalk density shown as green surface and tRNA density shown as beige surface). The hibernation factor Lso2 is shown as a green ribbon, occupying the tRNA P- site in **e**. **g, h** Close-up views of hibernation factor Lso2 from the *P. locustae* structure.

Interestingly, in our structure of the isolated 70S ribosome, we did not observe densities that could account for the hibernation factors seen in the previously determined single-particle structures of microsporidian ribosomes from *V.* *necatrix*, *P.* *locustae* and *E.* *cuniculi*. No densities suggesting the presence of MDF1 (refs. ^16,18^) or Lso2 (ref. ^17^) were evident in the A, P or E sites. In addition, the polypeptide exit tunnel did not contain a blocking hibernation factor, such as MDF2 or Lso2 (Extended Data Fig. 6). Multiple rounds of 3D classification did not reveal any variability in this region.

On the basis of these observations, we investigated which hibernation factors are present in and transcribed from the genome of *S.* *lophii*. Searches of the *S.* *lophii* genomes using BLASTP and TBLASTN for known microsporidian hibernation factors showed that homologues of MDF1 and Lso2 are encoded in the genome, while MDF2 could not be detected (Supplementary Fig. 11). MDF2 is a protein that has only been identified in *V.* *necatrix*, *N.* *ceranae* and *N.* *apis*, and it is possible that it is either an innovation within the *Vairimorpha* and *Nosema* lineage, or that homologues are present but hard to detect in more distantly related species. Interrogating RNA sequencing data of *S.* *lophii* detected reads that could be mapped to *mdf1* and *lso2*, suggesting that both genes are expressed. Curiously however, read coverage did not stretch to the 5′ end of the *lso2* gene but started 17 nucleotides into the predicted gene (Supplementary Fig. 11). Mass spectrometry analysis of our *S.* *lophii* samples revealed that MDF1 was present in all fractions of the ribosome purification (Supplementary Fig. 12).

**Extended Data Fig. 7:**
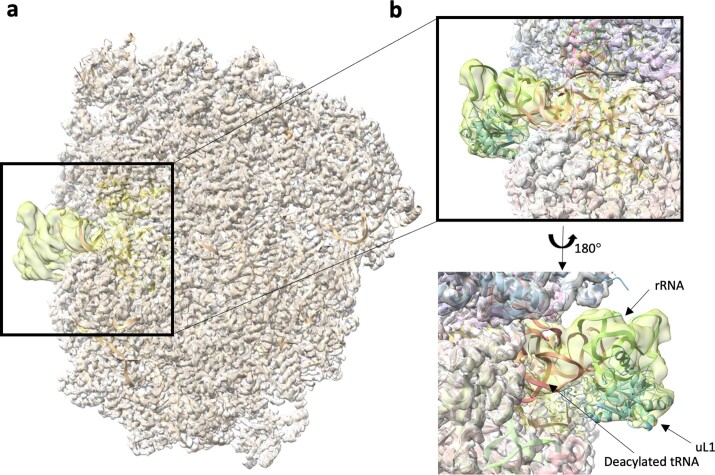
Atomic model of the L1 stalk of the isolated 70S ribosome. **a** The density map of the whole ribosome is shown as a transparent grey surface with the model as peach ribbons. The map of the L1 stalk (filtered with a B factor of 200 Å^2^) is shown as a transparent yellow surface. **b** A close up of the L1 stalk showing the modelled rRNA as a green ribbon, protein uL1 as a cyan ribbon and a deacylated tRNA in the ribosome E site as an orange ribbon.

Instead of harbouring previously described hibernation factors, our single-particle maps show a different, weak density near the ribosome’s E site (Extended Data Fig. 7). Focused classification and refinement of this part of our single-particle map resulted in a map with local resolution ranging between 3 and 6 Å (Extended Data Fig. 3c). This map enabled us to identify this density as the L1 stalk in closed conformation, which is bound to a tRNA located in the E site of the ribosome (Fig. 2b,c and Extended Data Fig. 7b). Further 3D classification did not reveal ribosomes that lacked the density for the E site tRNA, suggesting that the tRNA was bound to the great majority of the ribosomes in the sample, but flexible. The rRNA and protein uL1 (predicted by Alphafold2) of the L1 stalk, and a deacylated tRNA were modelled into this map (Fig. 2b,c and Extended Data Fig. 7). The tRNA shows three distinct interactions with the ribosome: (1) the 3′ CCA terminus on the acceptor arm interacts with protein eL42 and the 23S rRNA, (2) the anticodon arm binds the 16S rRNA and protein uS7 and (3) the elbow of the tRNA interacts with the L1 loop of the LSU rRNA and uL1 protein, which make up the L1 stalk. This is reminiscent of the structure of the hibernating ribosome dimer from *E.* *coli*, where a deacetylated tRNA was found in the E site^9^. However, while the tRNA was stabilized by the hibernation factor HPF in the *E.* *coli* ribosome dimer, a similar protein is not observed in our structure.

## A unique ribosomal dimerization mechanism

**Fig. 3:**
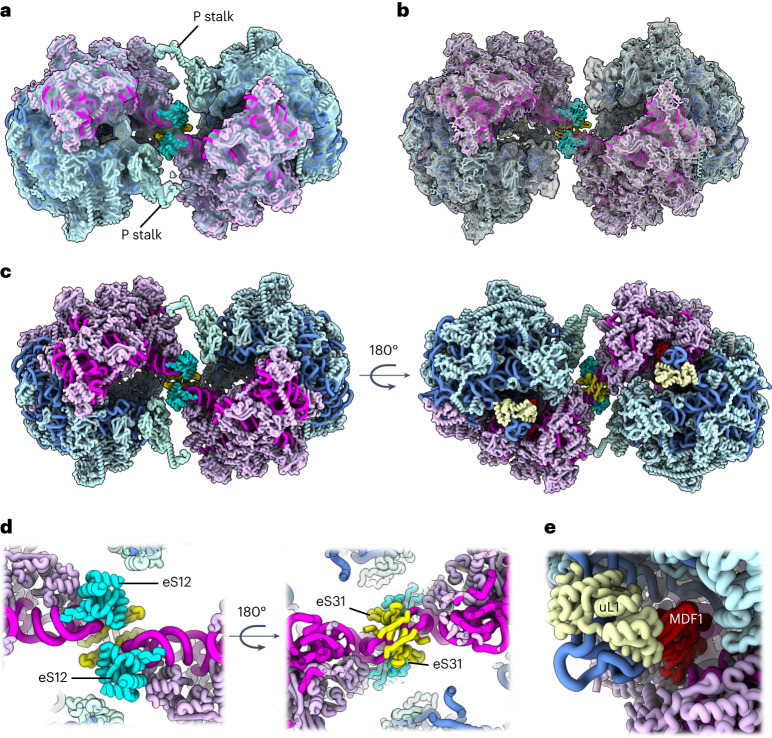
Map and model of the hibernating ribosome dimer from *S. lophii*. **a**, The ribosome dimer map at 14.3 Å resolution determined by subtomogram averaging (transparent grey) with atomic model of the dimer fitted (licorice representation). **b**, Two individual half-dimer maps at 10.8 Å resolution (transparent grey) superimposed with the dimer map (from **a**) and the atomic model fitted. Note that the P stalks are better defined in **a**, while the map has more detail in **b**. **c**, Two views of the atomic model of the dimer shown without the map and rotated by 180°. LSU rRNA, blue; LSU protein, light blue; SSU rRNA, magenta; SSU protein, light pink; L1 protein, cream. **d**, Two close-ups of the dimer interface, rotated by 180°. The dimer interface appears to be established by the subunits eS12 (cyan) and eS31 (yellow). **e**, Close-up of the E site. uL1, cream; MDF1, red.

**Fig. 4:**
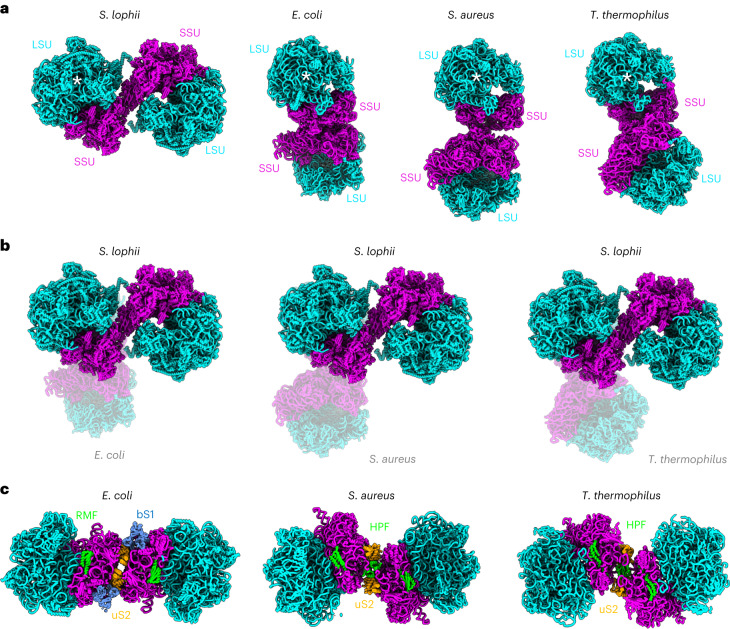
Distinct dimer architecture in eukaryotes and bacteria. **a**, Atomic models of *S.* *lophii* and various bacterial species shown side by side (licorice representation). The 70S ribosomes (half dimers) indicated by a white star (*) are in the same orientation. **b**, Hibernating ribosomes from three bacterial species (transparent) superimposed with that of *S.* *lophii* (opaque), highlighting distinct dimer architectures. **c**, Bacterial dimer interfaces with key subunits highlighted. Note that while in *S.* *lophii* and bacteria the dimer interface is established via the small ribosomal subunit, the exact location differs. In *S.* *lophii*, the dimer interface is established via the ribosomes’ beaks, while it is formed near uS2 in bacteria and mediated by the hibernation factors RMF/bS1 in *E.* *coli* and HPF in *S.* *aureus* and *T.* *thermophilus*.

To obtain a detailed structure of the eukaryotic hibernating ribosome dimer, we fitted our atomic model (determined by single-particle analysis) into our dimer map obtained by subtomogram averaging (Fig. 3a,b). This revealed a 100S particle that adopts a conformation that is entirely different compared with its bacterial counterparts (Fig. 4). Even though the orientations of the 70S ribosomes within the 100S dimers from *E.* *coli*, *Staphylococcus aureus and T.* *thermophilus* differ by a rotation around the dimer interface^22^, the dimer contact is always established between the subunits uS2 (via RMF in *E.* *coli* and HPF in *S.* *aureus and T.* *thermophilus*) (Fig. 4). In contrast, the eukaryotic dimer interface observed in *S.* *lophii* is located between proteins eS31, eS12 and the 16S rRNA in the beak of the SSU (Fig. 3d), within a connecting bridge between the two ribosomes in the dimer (Fig. 3a). This suggests an entirely different mechanism of dimer formation to those seen in bacteria. In line with this, no homologues to known bacterial dimerization factors were identified from our mass spectrometry or genomic sequence analyses.

**Fig. 5:**
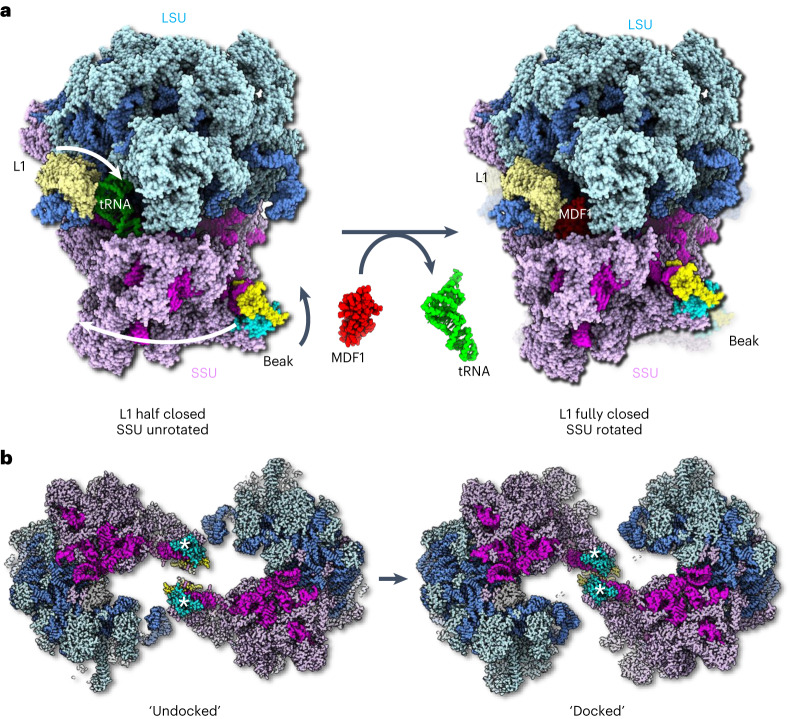
Conformational differences between the tRNA-bound 70S ribosome and the MDF1-bound hibernating 100S ribosome. **a**, Left: the structure of the (uninhibited) 70S ribosome in atom representation. The L1 stalk (blue, cream) is bound to an E-site tRNA (lime green) and is in a ‘half-closed'’ conformation. The small ribosomal subunit (SSU, magenta and pink) is in an unrotated state. The beak is in a straightened position. Right: atomic model of a half-dimer within a hibernating 100S particle. The E-site tRNA is replaced with the putative MDF1 protein (red). The L1 stalk is in a ‘fully-closed’ conformation and the SSU is in a rotated state. The beak is bent towards the large ribosomal subunit (LSU, shades of blue). Arrows in the left panel indicate conformational changes of L1, SSU and beak as the ribosome transitions from tRNA bound to MDF1 bound. **b**, Left: the tRNA-bound structure superimposed with the subtomogram averaging map of the 100S dimer shows a gap between the beaks (*). The beaks are in an ‘undocked’ conformation. Right: atomic model of the 100S ribosome dimer. Rotation of the SSU and bending of the beaks establishes close contact between the beaks, which are now in a 'docked' conformation. An animated version of this figure can be found in Supplementary Video 1.

**Extended Data Fig. 8:**
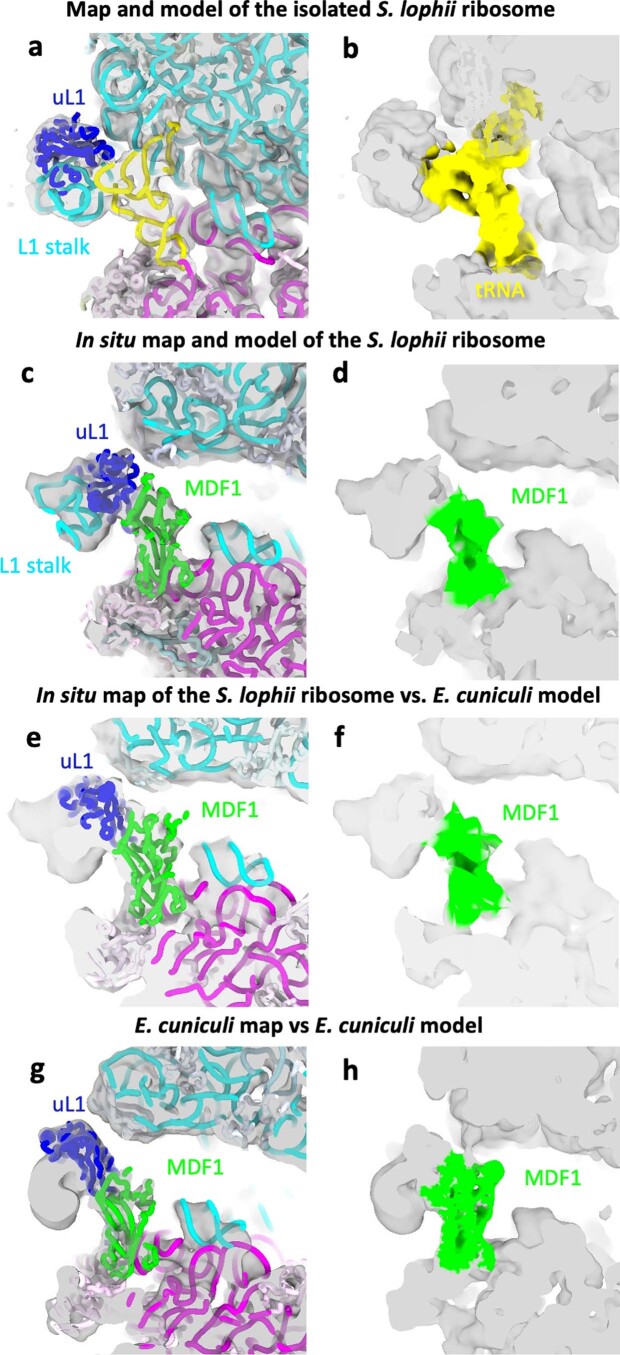
Putative MDF1 bound to the hibernating ribosome dimer of *S. lophii*. **a** Close-up of the L1 stalk region and E-site of a multibody single particle map of the isolated 70S ribosome from *S. lophii* with the atomic model fitted (multicolour, liquorice representation). **b** shows the same region highlighting the map region corresponding to the tRNA in yellow. The tRNA density shows a clear T-shape. **c, d** *In situ* subtomogram average of the hibernating 100S ribosome of *S. lophii* (half-dimer map) with the atomic model fitted in **c** and the map region occupied by the putative MDF1 colored green in **d**. The density assigned to MDF1 fits the dimensions and shape of the molecule. The density also clearly lacks the tRNA acceptor arm seen in the single particle map of the isolated 70S ribosome (**a, b**). **e** Atomic model of the *E. cuniculi* 70S hibernating ribosome (7QEP^18^) fitted into the *in situ* map of the *S. lophii* hibernating 100S ribosome (half dimer map). **f** shows the map region overlapping with MDF1 in green. The model of *E. cuniculi’s* MDF1 fits the location, size and shape of the *S. lophii* density. **g** Map of the hibernating 70S ribosome from *E. cuniculi* (EMD-13936) with the corresponding atomic model of *E. cuniculi* (7QEP) fitted, and the map region corresponding to MDF1 coloured in green (**h**). The dimensions and shape of the map region assigned to MDF1 is almost identical to that within the hibernating 100S ribosome of *S. lophii* (**c** & **d**). All single particle maps have been filtered to a resolution comparable to that of the subtomogram average. The coloured map regions in **b**, **d**, **f** and **h** have been produced using the color zone command in ChimeraX.

**Extended Data Fig. 9:**
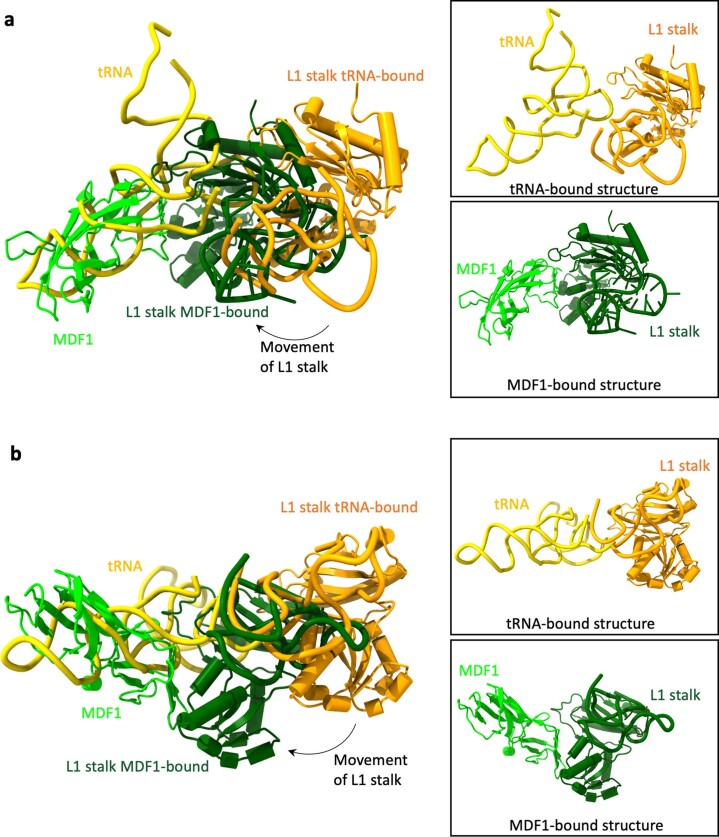
Comparison of the *S. lophii* L1 stalk position in the tRNA-bound and MDF1-bound states. **a**, **b** Two different views of the *S. lophii* L1 stalk in an overlay of the tRNA-bound and MDF1-bound states. tRNA shown in yellow with corresponding L1 stalk (consisting of the uL1 protein and rRNA) in orange (from the monomeric *S. lophii* ribosome structure, 7QCA). MDF1 in lime with corresponding L1 stalk (consisting of the uL1 protein and rRNA) in dark green (from the dimeric *S. lophii* ribosome structure, 8P5D). Boxed figures show the aligned tRNA-bound and MDF1-bound states alone for each view to clarify the difference observed between the positions of the L1 stalk.

In contrast to our single-particle map, our subtomogram averages of the dimer did not suggest the presence of an E-site tRNA, lacking density for the acceptor arm of the tRNA molecule. Instead, this region was occupied by a density that was similar to MDF1, as observed in the *E.* *cuniculi* ribosome^18^ (Extended Data Fig. 8). Superimposing the Alphafold2 prediction of *S.* *lophii* MDF1 matched the density and was corroborated by our mass spectrometry data. In this position, MDF1 would mimic an E-site tRNA, as previously suggested (Fig. 3c,e and Extended Data Fig. 8)^18^. When comparing our single-particle structure with the subtomogram average, we find that the L1 stalk, when bound to the proposed MDF1 protein, is closed even further than when tRNA bound (Fig. 5a and Extended Data Figs. 8,9). This indicates that the proposed MDF1 would hold the L1 stalk in a conformation that is incompatible with tRNA binding. However, due to the limited resolution of our in situ map, there remains some ambiguity regarding the molecular assignment of this region. Future work yielding near-atomic resolution maps will be required to unambiguously confirm the identity of the E-site density and the exact structure of the L1 stalk.

Interestingly, the conformational differences between the tRNA-bound 70S ribosome and MDF1-bound hibernating dimer were not limited to the L1 stalk. Close inspection of the 100S density in comparison with the 70S model revealed that the ribosomes within the hibernating dimer are in their rotated state, in contrast to the unrotated 70S ribosome (Fig. 5a). In addition, the beaks of the dimer-borne ribosomes are bent towards the large ribosomal subunit (Fig. 5b), in a similar way to the structure of the MDF1-bound 70S ribosome isolated from *E.* *cuniculi*^18^. This movement of the beaks closes the gap that would otherwise exist between the ribosomes in the observed 100S configuration, thus transitioning the dimer from an ‘undocked’ to a ‘docked’ conformation (Fig. 5b). The conformational changes between the tRNA-bound 70S and MDF1-bound 100S ribosome are shown as a morph in Supplementary Video 1.

**Extended Data Fig. 10:**
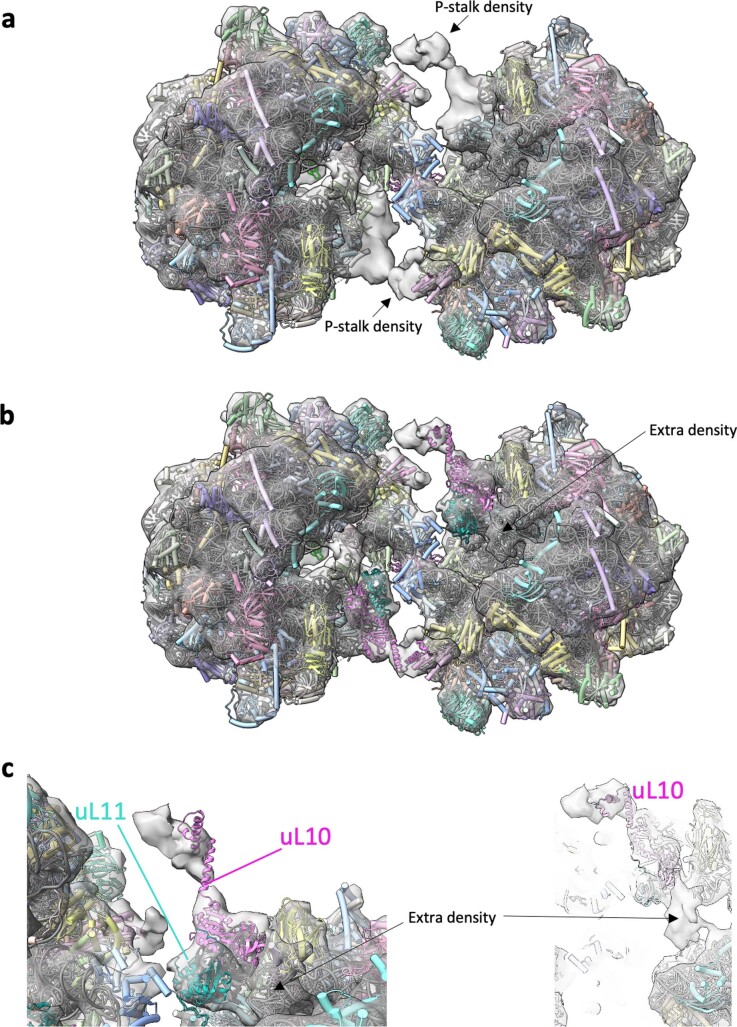
The P-stalk density of the 100S*S. lophii* ribosome. **a** *S. lophii* dimer model (PDB 8P60) (cylinders and stubs) and 14.3 Å map (transparent grey; EMD-17457) showing additional density for the P-stalk (black arrows). **b**, **c** Alphafold2 models of the *S. lophii* proteins uL10 (pink ribbon) and uL11 (cyan ribbon) were modelled into the dimer map guided by the structure of the porcine ribosome (3J7P^23^) - one of the few ribosome structures with a partially ordered P-stalk. An area of extra density was observed in a region where elongation factors have been observed to bind, but the density was not clear enough to model any proteins.

Furthermore, our dimer map showed a clear density for the P stalk (Fig. 3a), which was not resolved in the single-particle structure. We were able to model the dimer-borne P stalk based on the structure of a porcine ribosome, 3J7P (ref. ^23^) and Alphafold models of the individual *S.* *lophii* components (Extended Data Fig. 10). In eukaryotes, this region contains the ribosomal subunits uL10, uL11 and P1/P2, with uL10 forming the base of the stalk to which a number of dimers of P1/P2 can bind, and we were able to model proteins uL10 and uL11 into the P-stalk density. This stalk region is important for the binding of translational GTPases, which catalyse various steps of translation^24^. An additional density was also observed near the P stalk (Extended Data Fig. 10) in a region where elongation factors have been observed to bind, but the density was not clear enough to model. In the 100S hibernating ribosome, the P stalk is located close to the dimer interface, where it may form interactions with beak proteins eS31 and eS12 from the opposite subunit of the dimer and thus stabilize the 100S complex (Fig. 3 and Extended Data Fig. 10).

## Discussion

Here, we report the in situ structure of a eukaryotic hibernating ribosome dimer from PTs of the microsporidian species *S.* *lophii*. The striking difference between the eukaryotic dimer from *S.* *lophii* and those from bacteria suggest different dimerization mechanisms in bacterial and eukaryotic 100S ribosomes. The observation that ribosomal dimerization is retained in the otherwise strongly reduced microsporidian ribosome highlights its importance for ribosomal hibernation and the protection of ribosomes during phases of cellular dormancy. An open question to be answered is how widespread and variable hibernating ribosome dimers are amongst eukaryotes. Interestingly, one study provided evidence that hibernating ribosome dimers may occur in rat glioma cells under amino acid starvation conditions^12^. This suggests the intriguing possibility that ribosomal dimerization is conserved throughout the eukaryotic kingdom, including mammals, with unforeseen implications for cellular homeostasis and health.

The dimer interface involves the ribosomal subunit eS31. This protein is one of two eukaryotic-specific ubiquitin fusion proteins found within the ribosome, the second one being eL40, which is situated at the base of the P stalk. These two ubiquitin fusion proteins are universally encoded in the genomes of model eukaryotes^25^. Ubiquitin is a reversible post-translational modifier and is involved in many different cellular processes, being added to, and removed from, proteins and consequently altering their localization or activity. In the ribosome, ubiquitin domains are themselves proteolytically removed from both eS31 and eL40 (ref. ^26^). A recent review questions whether the selective advantage of ubiquitin being fused to eS31 and eL40 could be a means of coupling the synthesis and degradation of proteins to maintain proteostasis in eukaryotes. It was also discussed that the specific fusions of ubiquitin with eS31 and eL40 over any other ribosomal proteins suggest an important role for ubiquitin in eS31 and eL40 expression, ribosome assembly or ribosome function^25^. Our observation that both eS31 and eL40 are involved in or close to the dimer interface may thus point to a role in ubiquitin processing in the regulation of ribosome hibernation and 100S formation in eukaryotes.

Moreover, eS31 and eL40 both contain a highly conserved zinc-binding motif (totally conserved in the top 100 blast hits), and we have modelled zinc into our single-particle structure (Extended Data Fig. 4d,e). Ribosome hibernation has been shown to occur in *Mycobacterium smegmatis* as a direct result of zinc starvation, with Mycobacteria and other bacteria remodelling their ribosomes in response to zinc depletion by replacing zinc-binding ribosomal proteins with zinc-free paralogues, and releasing zinc for other metabolic processes^27,28^. The authors proposed that ribosome hibernation is a specific and conserved response to zinc depletion in mycobacteria. The presence of the highly conserved zinc-binding eS31 protein at the dimerization interface raises the intriguing potential of zinc binding playing a role in eukaryotic ribosome dimerization in response to stress. Taken together, eS31 and possibly eL40 may be important signalling hubs for the initiation and termination of ribosomal hibernation, and mediators for the formation of 100S ribosomes.

The P stalk has previously been shown to increase the local concentration of the translational guanosine exchange factors EF1A and EF2, while flexibly moving on the ribosome, hence promoting polypeptide elongation^29^. In the hibernating ribosome dimer, the P stalk is orientated towards, and may even participate in, the dimer interface. This appears to lock the usually flexible P stalks into a fixed position—evidenced by the fact that it was resolved in our dimer map, as opposed to most single-particle structures of individual ribosomes (including ours). It is likely that the static position of the P stalk would reduce its ability to exchange EF1A and EF2, resulting in the halting of translation.

Previous studies of purified hibernating ribosomes from the microsporidian species *P.* *locustae*, *V.* *necatrix* and *E.* *cuniculi*^16–18^ did not reveal dimers. This suggests that either ribosome dimers do not exist in those species or that, consistent with our single-particle data, the dimer interface is fragile and easily disrupted during sample preparation. Our cryoET data, combined with mass spectrometry, provide evidence of the presence of the hibernation factor MDF1 in the ribosome dimer, which is exchanged by a likely deacetylated E-site tRNA in the purified ribosome. The proposed MDF1 protein appears to cause a more complete closure of the L1 stalk when compared with the monomeric structure with deacylated tRNA in the E site. In these conditions, the ribosome is rendered inaccessible for mRNA and tRNAs. In contrast, neither our ribosome dimer structure, nor that of the monomer reveal the presence of a factor that blocks the polypeptide exit tunnel, as is the case for *P.* *locustae* and *V.* *necatrix*. This is consistent with the absence of MDF2 in the *S.* *lophii* genome, and suggests that blocking the polypeptide exit tunnel is not an essential requirement for ribosome hibernation, at least in some species.

The hibernating ribosome dimer was found to be in a rotated state, as opposed to the tRNA-bound 70S particle. Furthermore, the beaks within the dimer are bent towards each other, thus allowing the dimer interface to be established. While it is tempting to speculate that these conformational changes are induced by the putative MDF1 protein, further research will be required to confirm this notion.

Taken together, we show that the microsporidian sporoplasm is densely packed with ribosome dimers that are dormant but otherwise completely assembled and translationally competent. This shows that microsporidian spores maintain their ribosomes in an inactive, yet primed state, ready to reactivate once a host cell has been infected. Once the microsporidian parasite has invaded a host cell, the 100S ribosome dimers must convert into 70S monomers and shed their hibernation factor (MDF1) to become fully translationally active. In principle, this may simply ensue through mechanical disruption of the aparently fragile dimer interface, as observed during our single-particle sample preparation, or due to a dilution effect as observed in *E.* *coli*, where a reduction in ribosome concentration leads to a dissociation of the dimer to monomeric ribosomes^30^. It could be envisaged that a dilution of microsporidian ribosomes takes place when the microsporidian sporoplasm leaves the confined environment of the PT and expands as it enters the host cell. However, it is also likely that a so-far unknown microsporidian or host-cell signal is required to activate the hibernating microsporidian ribosomes. Further studies will need to be undertaken to investigate the exact sequence of events that take place during eukaryotic ribosomal hibernation and activation in microsporidia and beyond.

## Methods

### Spore preparation

To isolate spores of *S.* *lophii*, clusters of cysts were collected from monkfish (*Lophius piscatorius)* caught in the North Atlantic and landed in Devon (United Kingdom). Xenomas filled with microsporidia spores were removed from fish tissue manually. Samples were homogenized manually using a scalpel in phosphate-buffered saline until a suspension was obtained. The suspension was filtered through a 100 µm mesh sterile cell strainer (Fisher Scientific). The spores were cleaned by centrifugation in a 25–50–75–100% Percoll gradient (Sigma) at 4 °C and 3,240*g* for 1 h. The spores were collected and washed three times in sterile phosphate-buffered solution. The sample was stored at 4 °C with the addition of 10 µg ml^−1^ of ampicillin. The concentration of spores in the solution was determined using a haemocytometer.

### Preparation of EM grids with germinated spores

*S.* *lophii* spores in suspension were mixed in 1:1 proportion with 10 nm protein A-gold (Aurion) as fiducials. Then, 3 µl of the mixture were deposited on glow-discharged R2/2 Copper 300 mesh holey carbon-coated support grids (Quantifoil) along with 1 µl of HEPES pH 10 and 1 µl of hydrogen peroxide 0.9%. The grids were initially screened and optimized in negative stain. For cryoET, grids were plunge-frozen in liquid ethane using a Vitrobot Mark-IV (Thermo Fisher) using variable blot times of 4–6 s and a blot force of −1.

The grids were screened using a 120 kV Tecnai Spirit (Thermo Fisher Scientific). Tilt series were collected across two separate sessions with 300 kV Titan Krios microscopes (Thermo Fisher Scientific) at the Electron Bioimaging Centre (eBIC). The first Krios was equipped with a K2 Summit direct electron detector and the second a K3 direct electron detector (Gatan). The tilt series were collected using the SerialEM software^31^ with pixel sizes of 4.377 Å and 4.53 Å (2.265 Å super resolution). A nominal defocus range of −3 to −6 µm was used across both datasets. A tilt range of −60° to +60° with 2° steps in a dose-symmetric tilt scheme^32^ was employed for data collection with a total target dose of 120 e Å^−2^. A total of 71 tomograms were collected, with 20 containing ribosomes that were used for subtomogram averaging.

### Tilt series reconstruction and subtomogram averaging

Motion correction and contrast transfer function estimation of movies were performed using Warp^33^. Tilt series were aligned using AreTomo^34^ and binned, deconvolved tomograms reconstructed in Warp. Particles were picked manually using IMOD^35^, then extracted as Fourier cropped (10 Å per pixel) subvolumes in Warp. A total of 6,505 particles were selected. Then, 3D classification steps and refinement of the monomers were performed in Relion^20^ until the Nyquist limit was reached. Particles were re-extracted at 6 Å pixel size and refined again to a resolution of 17.5 Å. The data were imported to M^21^ and refined, improving the resolution to 12.6 Å. Particles were again re-extracted at 4.53 Å per pixel and refined in Relion. After duplicate removal, further refinement of 2,177 half-dimer particles in Relion achieved a final resolution of 10.8 Å/15.5 Å/11.6 Å at FSC 0.143/FSC 0.5/map/model FSC (Supplementary Figs. 3 and 13).

To resolve the entire dimer, the previously refined particles were extracted with Fourier cropping and a larger box size to encompass the whole dimer. Particles were classified, removing poor quality particles and monomers. Duplicate particles within 250 Å were removed. Particles were aligned and re-extracted in M with the centre point defined as the middle of the pair. Refinements were performed in Relion with 1,191 particles, using C2 symmetry. The final map had a resolution of 14.3 Å/20 Å/18.2 Å at FSC 0.143/FSC 0.5/map/model FSC (Supplementary Figs. 3 and 13). See Supplementary Table 1 for a summary of the cryoET data collection and associated Electron Microscopy Data Bank (EMDB) and Protein Data Bank (PDB) depositions. The subtomogram averaging pipeline is shown in Supplementary Fig. 2 and the cryoET statistics and deposition details are presented in Supplementary Table 1.

### Ribosome purification

*S.* *lophii* spores were washed and resuspended in sterile BS100 buffer (25 mM HEPES, 100 mM potassium acetate, 15 mM magnesium acetate and 1 mM DTT). Spores were disrupted using a Fastprep-24 5 G bead beater (MP Biomedicals, Fisher Scientific) and 0.1 mm glass beads. Cell debris and non-disrupted spores were pelleted by centrifugation at 9,000*g* for 15 min at 4 °C. The supernatant was collected and layered on top of a 30% sucrose cushion in BS100 buffer and centrifuged at 206,000*g* for 3 h at 4 °C. The supernatant was discarded, and the pellet was resuspended by gentle shaking for 1 h at 4 °C in BS100 buffer. The crude extract was layered over a continuous sucrose gradient (10–40%) in BS100 buffer and centrifuged at 125,000*g* for 80 min at 4 °C. Fractions were collected and analysed for RNA using a Nanodrop (Thermo Scientific) at 260 nm. Vivaspin columns (Sartorius) with 100 kDa cut-off were used to remove the excess of sucrose and concentrate the sample to a concentration between 5 and 15 mg ml^−1^.

### Single-particle cryoEM

Three microlitres of purified ribosome sample were applied to Quantifoil 300 mesh R2/2 grids with an ultrathin 2 nm carbon layer and frozen in liquid ethane using a Vitrobot Mark IV (Thermo Fisher Scientific) at 15 °C, 100% relative humidity, blot force −1 and blot time 4 s.

Three datasets were collected on a 200 kV Talos Arctica microscope (Thermo Fisher Scientific) with a K2 Summit direct electron detector at the Regional Facility for High-Resolution CryoEM, University of Bristol, UK. Datasets were collected using the EPU software (Thermo Fisher Scientific) with pixel sizes of 1.054 Å, two in counting mode and one in super resolution (0.525 Å) with a defocus range of −0.8 μm to −2 μm. A final dataset was collected on a 300 kV Titan Krios microscope with a K3 direct electron detector at eBIC. The data were collected using EPU with a pixel size of 1.06 Å (0.53 Å super resolution).

Motion correction, contrast transfer function estimation and particle picking were performed using Warp^33^. A total of 977,879 particles were picked from 26,997 micrographs. All four datasets were initially processed separately and combined for the later refinement steps. Several iterations of two-dimensional and 3D classification and refinement were performed in Relion 3.1 (ref. ^20^). The two maps used for model building were generated by a three-body refinement of the large subunit, SSU body and SSU head (285,940 particles) and a refinement of the best class of a 3D classification with no alignment. The resolutions obtained for these maps were 2.26 Å, 2.48 Å and 2.86 Å for LSU, SSU body and SSU head, respectively, and 2.78 Å for the whole-ribosome map (2.4 Å according to Map/Model FSC). The L1 stalk was resolved using a tight mask around the region, with a 3D classification with no alignment. The best class was then refined to a global resolution of 3.07 Å. The local resolution range for this region was 3–6 Å, with the density of L1 stalk and tRNA having a resolution of 5–6 Å. Postprocessing of the maps was performed using DeepEMhancer^36^. The single-particle analysis pipeline is shown in Supplementary Fig. 7.

### Model building, refinement and validation

A model of the *V.* *necatrix* ribosome (pdb 6RM3 (ref. ^16^)) was used as a starting template to build each individual protein and RNA chain into the single-particle reconstruction maps using Coot^37^. The individual maps were superimposed onto a reference whole-ribosome map using Chimera^38^ and the CCP4 software suite^39^. Individual chains were positioned into the *S.* *lophii* ribosome map using Chimera, Coot or the phased molecular replacement option in MOLREP (ref. ^40^) adapted for use in cryoEM^41^. The density maps were of high quality and allowed to build the full atomic model for most of the rRNA and of 71 proteins, in many cases extending the chains that were present in the *V.* *necatrix* template model. CryoEM density peaks within 2.0–2.1 Å of oxygen ligands were modelled as Mg^2+^ ions (Extended Data Fig. 4b). Peaks with both oxygen and nitrogen ligands and metal-ligand distances exceeding 2.6 Å were modelled as potassium ions (Extended Data Fig. 4c), these were in line with potassium ion assignment by long-wavelength X-ray crystallography in the *T. thermophilus* ribosome^42^. The residues of the C-terminal part (77–103) of the protein eL24 that were missing in the *V.* *necatrix* structure (pdb 6RM3 (ref. ^16^)) were built as a polyalanine model. Later, a full atomic model of eL24 was predicted using the Alphafold2 software^43^, which allowed sequence assignment for residues of the polyalanine model. Additionally, the Alphafold2 prediction of the C-terminal (non-containing Ub) part of the eS31 protein was successfully positioned in the SSU head density map with addition of Zn^2+^ ion (Extended Data Fig. 4d,e). Each protein model was rebuilt and validated using the ISOLDE software^44^, implemented in ChimeraX^45^. The model was refined using REFMAC5 (ref. ^46^) from the CCPEM package^47^. The structure of *S.* *lophii* uL1 was predicted with Alphafold2 and the position of it obtained within the L1 stalk map by aligning 5T62 from *S.* *cerevisiae*^48^. The deacylated tRNA in the E site was obtained by overlaying structure 6H4N from *E.* *coli* as a starting reference^9^.

Two *S.* *lophii* ribosome particles were fitted into the subtomogram average using ChimeraX^45^. Close inspection of the maps revealed that some SSU proteins did not fit the density well and this corresponded to the ribosomes used as models being in non-rotated conformations. The SSU was modelled in its rotated position with the aid of an overlaid structure of the *E.* *cuniculi* ribosome (7QEP (ref. ^18^)). The L1 stalk, P stalk and MDF1 were modelled into the additional density observed in the dimer map, using Alphafold2 to model individual proteins. For a summary of the single-particle data collection, associated model building parameters, and EMDB and PDB deposition codes, see Supplementary Table 2.

### Nanoscale liquid chromatography mass spectrometry

Samples were run on a 10% sodium dodecyl sulfatepolyacrylamide gel electrophoresis gel until the dye front had migrated approximately 1 cm into the separating gel. Each gel lane was then excised as a single slice and subjected to in-gel tryptic digestion using a DigestPro automated digestion unit (Intavis Ltd.).

The resulting peptides were fractionated using an Ultimate 3000 nano-LC system in line with an Orbitrap Fusion Tribrid mass spectrometer (Thermo Scientific). In brief, peptides in 1% (vol/vol) formic acid were injected onto an Acclaim PepMap C18 nano-trap column (Thermo Scientific). After washing with 0.5% (vol/vol) acetonitrile 0.1% (vol/vol) formic acid, peptides were resolved on a 250 mm × 75 μm Acclaim PepMap C18 reverse phase analytical column (Thermo Scientific) over a 150 min organic gradient, using seven gradient segments (1–6% solvent B over 1 min, 6–15% B over 58 min, 15–32% B over 58 min, 32–40% B over 5 min, 40–90% B over 1 min, held at 90% B for 6 min and then reduced to 1% B over 1 min.) with a flow rate of 300 nl min^−1^. Solvent A was 0.1% formic acid and Solvent B was aqueous 80% acetonitrile in 0.1% formic acid. Peptides were ionized by nano-electrospray ionization at 2.2 kV using a stainless-steel emitter with an internal diameter of 30 μm (Thermo Scientific) and a capillary temperature of 275 °C.

All spectra were acquired using an Orbitrap Fusion Tribrid mass spectrometer controlled by Xcalibur 2.1 software (Thermo Scientific) and operated in data-dependent acquisition mode. Fourier Transform Mass Spectrometry 1 spectra were collected at a resolution of 120,000 over a scan range (*m*/*z*) of 350–1,550, with an automatic gain control target of 400,000 and a maximum injection time of 100 ms. Precursors were filtered according to charge state (to include charge states 2–7), with monoisotopic peak determination set to peptide and using an intensity range from 5E3 to 1E20. Previously interrogated precursors were excluded using a dynamic window (40S ± 10 ppm). The MS2 precursors were isolated with a quadrupole mass filter set to a width of 1.6 *m*/*z*. ITMS2 spectra were collected with an automatic gain control target of 5,000, maximum injection time of 50 ms and HCD collision energy of 35%.

The raw data files were processed and quantified using Proteome Discoverer software v2.1 (Thermo Scientific) and searched against the UniProt *S.* *lophii* (1358809) database (downloaded November 2020, 2,499 sequences) using the SEQUEST HT algorithm. Peptide precursor mass tolerance was set at 10 ppm, and tandem mass spectrometry tolerance was set at 0.6 Da. Search criteria included oxidation of methionine (+15.995 Da), acetylation of the protein N-terminus (+42.011 Da) and methionine loss plus acetylation of the protein N-terminus (−89.03 Da) as variable modifications and carbamidomethylation of cysteine (+57.021 Da) as a fixed modification. Searches were performed with full tryptic digestion and a maximum of two missed cleavages were allowed. The reverse database search option was enabled and all data were filtered to satisfy a false discovery rate of 5%. Raw data files were also searched against a hypothetical translation of the Celtic Deep (GCA_001887945.1, translated by getorf, minimum 60 nucleotides) to identify any short hypothetical genes not annotated in the *S.* *lophii* genome project.

### Sequence analysis

rRNA genes were retrieved from whole-genome shotgun sequence and Transcriptome Shotgun Assembly records (MQSS01000307.1 corrected with Transcriptome Shotgun Assembly sequence GALE01012333, MQSS01000329.1 and GALE01012333.1). Ribosomal protein sequences were retrieved from the *Spraguea lophii* genome Bioproject AMN02141961 (ref. ^49^). Not all ribosomal proteins were annotated in this original genome sequence due to the fact that the assembly was fragmented, some shorter ribosomal proteins were missed in the annotation, and the fact that some of them have short, rarely spliced introns that interrupt the open reading frames^50,51^. To find the full set of ribosomal proteins in *S.* *lophii*, the assemblies of ten environmental samples of *Spraguea* were searched using tblastn^49^. For poorly conserved proteins such as MDF2, msL1 and Lso2 that were not retrieved by this approach, Position-Specific Iterated Basic Local Alignment Search Tool and grep searches of conserved motifs in a predicted translation of the assembly in all three frames were used to look for evidence of their presence in the *Spraguea* genomes.

### Reporting summary

Further information on research design is available in the Nature Portfolio Reporting Summary linked to this article.

### Supplementary information

Supplementary Information Supplementary Figs. 1–13 and Tables 1 and 2.

Reporting Summary

Supplementary Video 1 An animated version of Fig. 5 showing the conformational differences between the tRNA-bound 70S ribosome and the MDF1-bound hibernating 100S ribosome.

## Data Availability

The atomic coordinates of the *S.* *lophii* ribosome monomer and the single-particle cryoEM maps were deposited to the PDB (https://www.rcsb.org) with accession number 7QCA and to the EMDB (https://www.ebi.ac.uk/emdb) with the accession number EMD-13892. The subtomogram average of the *S.* *lophii* ribosome dimer and the coordinates of two ribosome particles fitted into it were deposited to the PDB with accession number 8P60 and EMD-17457. The subtomogram average of one half of the *S.* *lophii* ribosome dimer and the coordinates of one ribosome particle fitted into it were deposited to the PDB with accession number 8P5D and EMD-17448. The mass spectrometry data have been deposited to the ProteomeXchange Consortium via the PRIDE partner repository (https://www.ebi.ac.uk/pride/) with the dataset identifier PXD04446, as well as to MicrosporidiaDB (microsporidiadb.org). The previously determined structures of ribosomes of *V.* *necatrix* (6RM3), *S.* *cerevisiae* (5T62, 3J77, 3J78), *S.* *scrofa* (3J7P), *E.* *coli* (6H4N), *E.* *cuniculi* (7QEP) and *P.* *locustae* (6ZU5) used in this study are available from the PDB under the accession codes listed.
