# Supplementary material for: CryoEM reveals that ribosomes in microsporidian spores are locked in a dimeric hibernating state
Source: Nat Microbiol. 2023 Sep 14;8(10):1834–45. doi: 10.1038/s41564-023-01469-w (PMC10522483; doi:10.1038/s41564-023-01469-w)
Supplement: Supplementary file 1 — Supplementary Figs. 1–13 and Tables 1 and 2. [file 41564_2023_1469_MOESM1_ESM.pdf]

nature microbiology

Article https://doi.org/10.1038/s41564-023-01469-w

CryoEM reveals that ribosomes in microsporidian spores are locked in a dimeric hibernating state

In the format provided by the authors and unedited

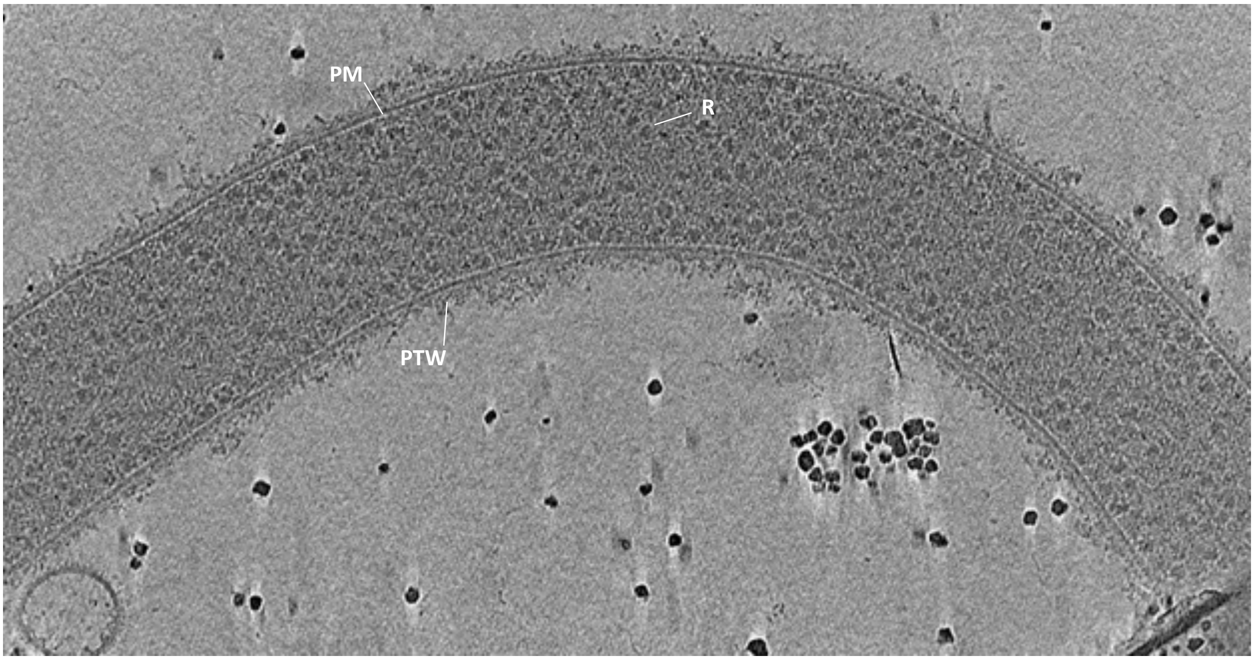

Supplementary Figure 1 – Slice through a tomogram of a polar tube of a germinated *S. lophii* spore.
PTW, polar tube wall, PM, plasma membrane, R, ribosome.

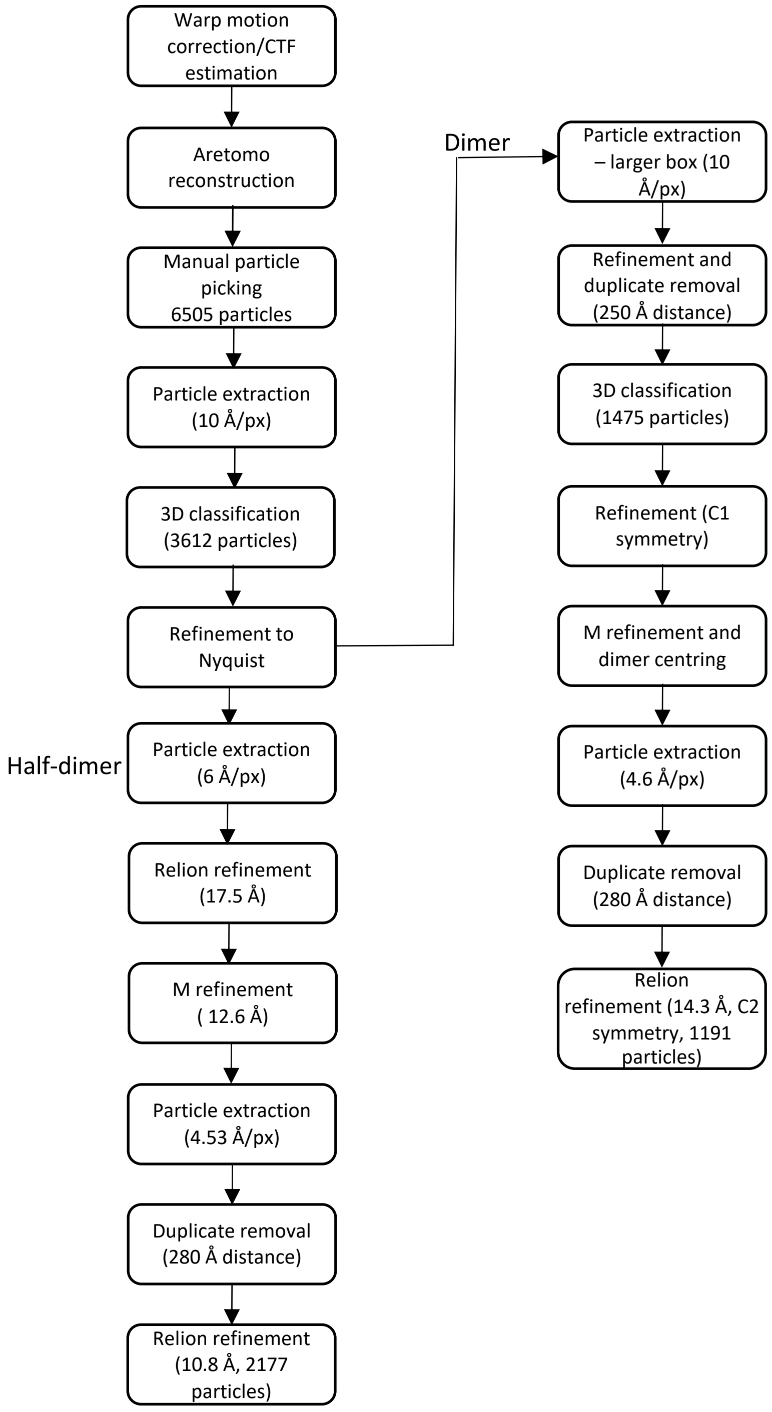

Supplementary Figure 2 - Flow chart of the subtomogram averaging pipeline.

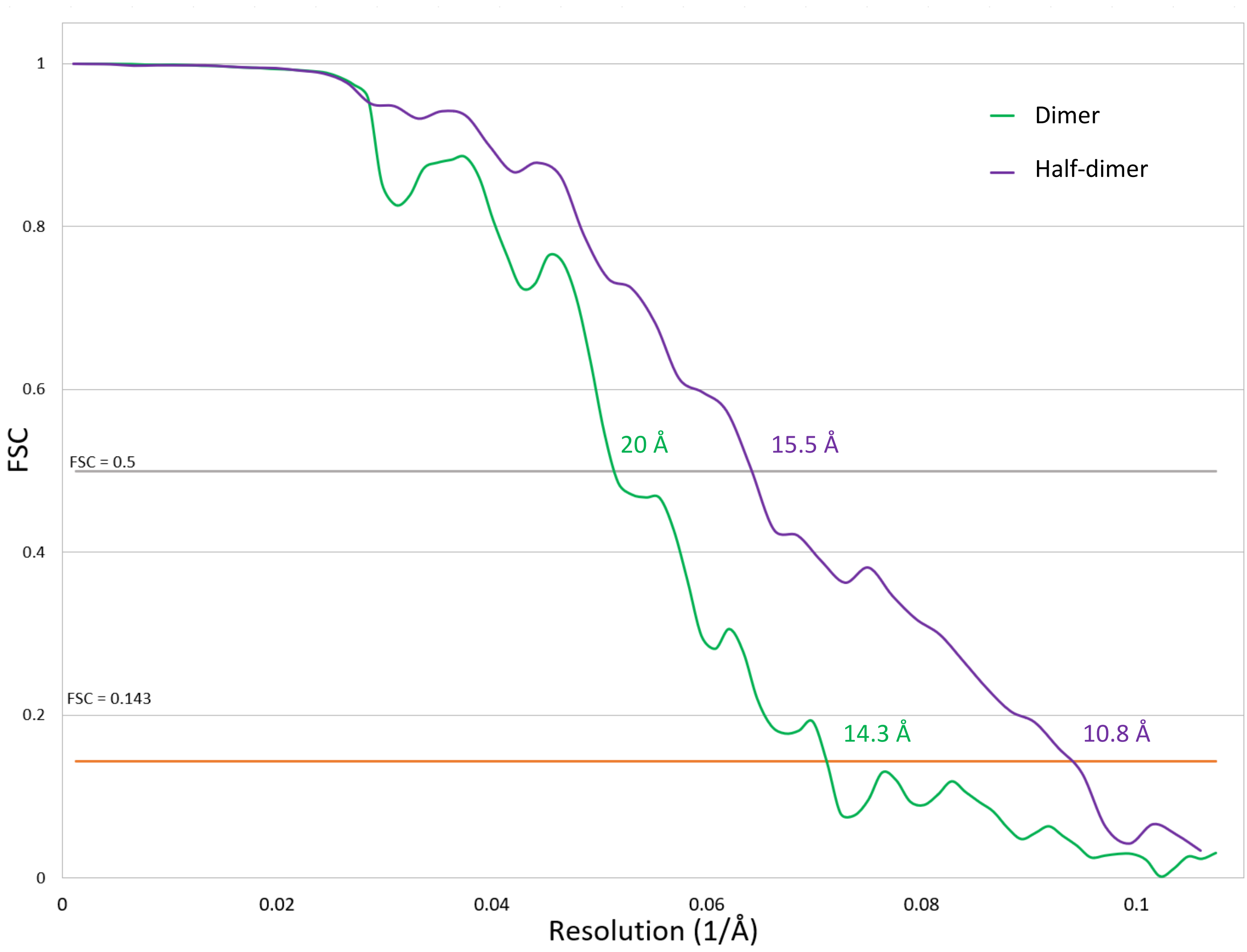

Supplementary Figure 3 - Resolution estimation of the *S. lophii* ribosome dimer and half-dimer maps from subtomogram averaging.
Gold Standard FSC of the dimer (green) and masked half-dimer within the average (purple).

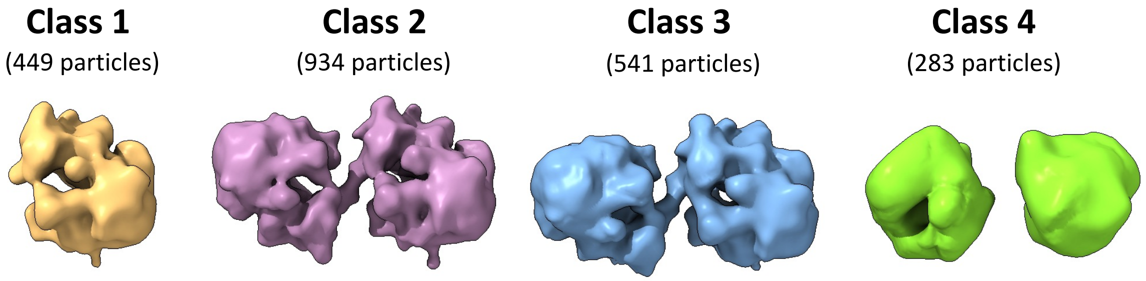

Supplementary Figure 4 – 3D classification of subtomogram average.
Out of a total of 2207 particles post duplicate removal, 449 particles contribute to a monomeric class (20%). In all 100 S dimers, the 70 S ribosomes adopt the same orientation with respect to each other. No obvious differences were observed between classes 2 and 3, and these were combined for further rounds of refinements.

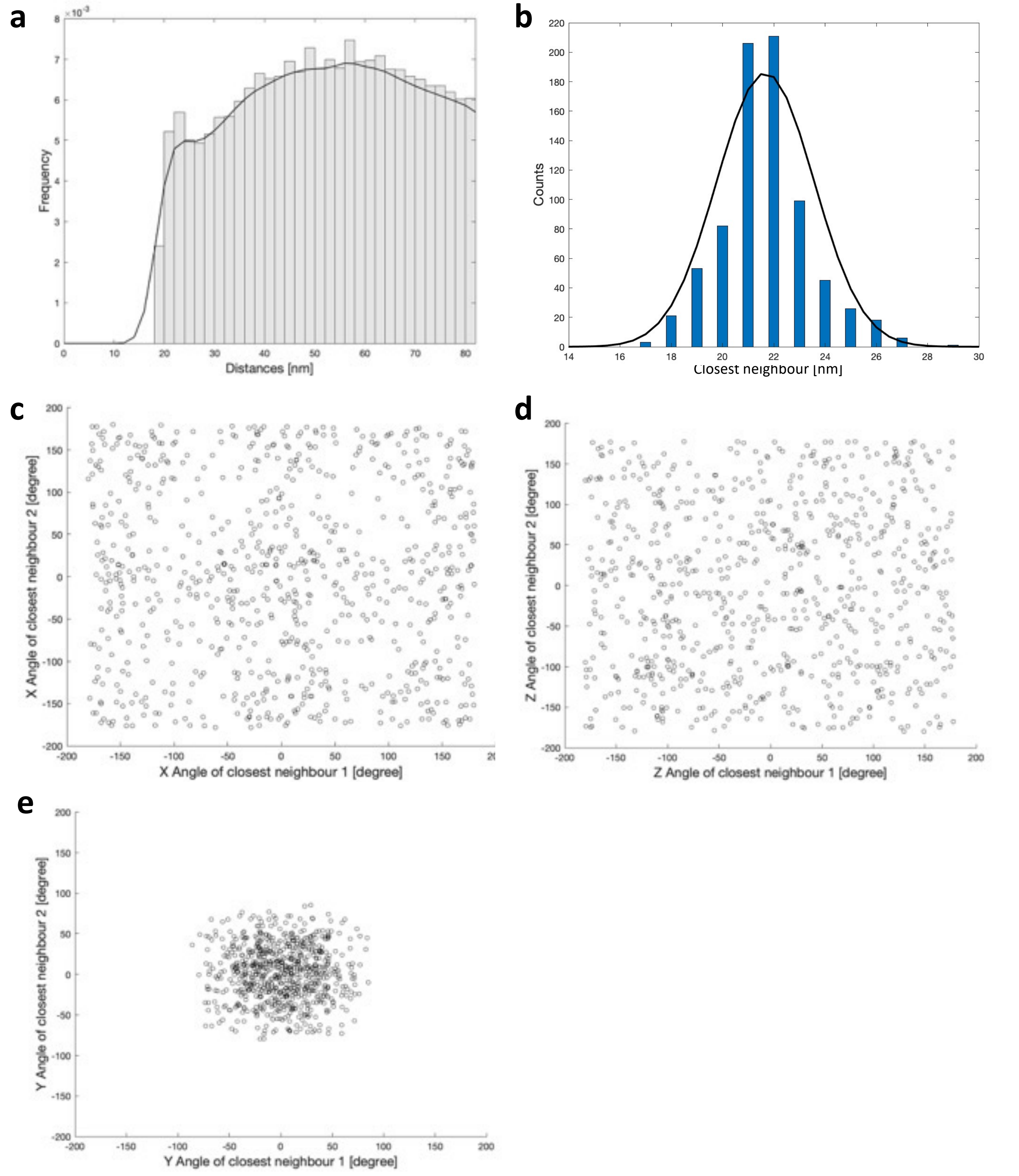

Supplementary Figure 5 – Statistical analysis of ribosome distribution within the polar tube.

a Histogram of distances between 70 S particles in a PT with a peak at around 21 nm, corresponding to the centre-to-centre distance between two partners in a dimer pair, or ribosomes in touching distance. No other peaks or multiples of the average distance between partners that could account for the presence of polysomes are evident. **b** Histogram showing only the distribution of distances between closest neighbours, with a peak at around 21 nm. **c-e** Angular relationships between closest 70 S neighbours of all particles. Scatter plots of the X and Z angles do not show any correlation and are randomly distributed between -180 and +180°. The Y angles of closest neighbours (i.e. dimers) of all particles range only between -90 and +90°, which is likely a manifestation of the relatively fixed angle between two 70 S particles within a dimer. The sample size was 771 ribosomes taken from one representative tomogram. Ribosomes were manually selected and subsequently aligned with an estimated accuracy of 10 Å, corresponding to the resolution achieved by sub-tomogram averaging.

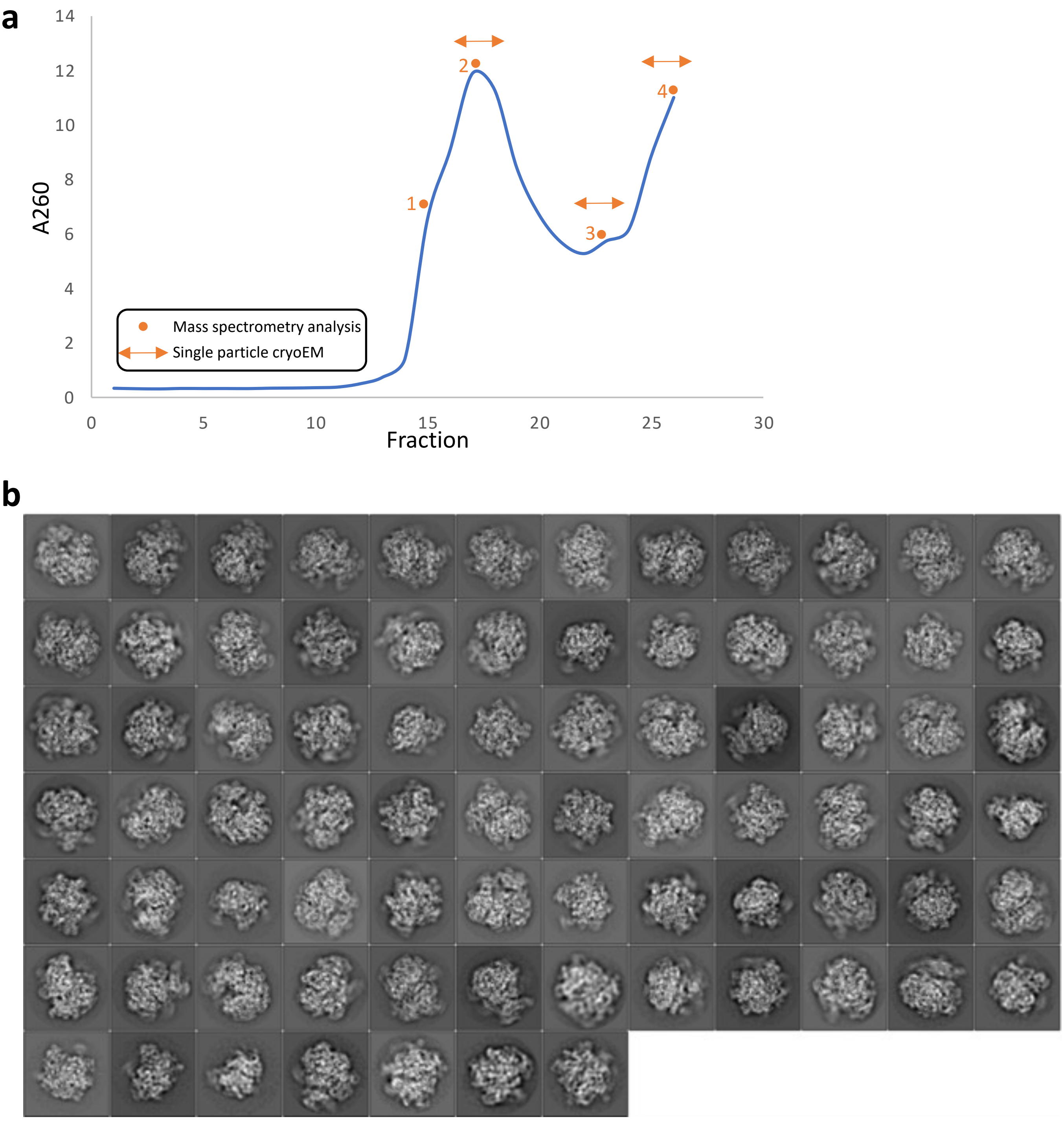

Supplementary Figure 6 – Purification profile of *S. lophii* ribosomes using a sucrose gradient.
a Purification profile. Samples taken for mass spectrometry analysis are marked with an orange dot, and the peaks investigated by cryoEM are shown with a double-headed arrow. **b** 2D classification of cleaned and polished single particle dataset.

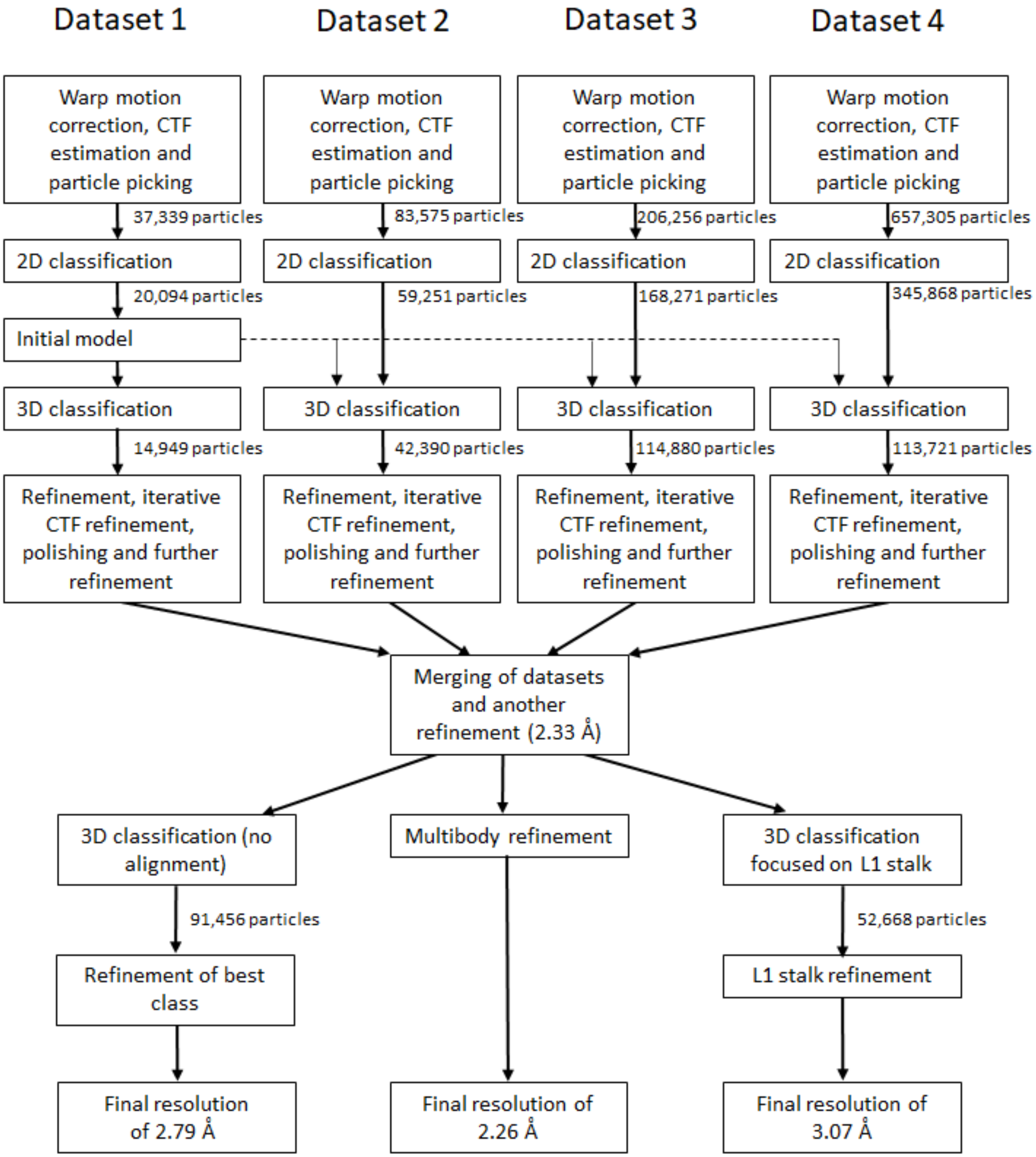

Supplementary Figure 7 – Flow chart of the single particle processing in Relion .

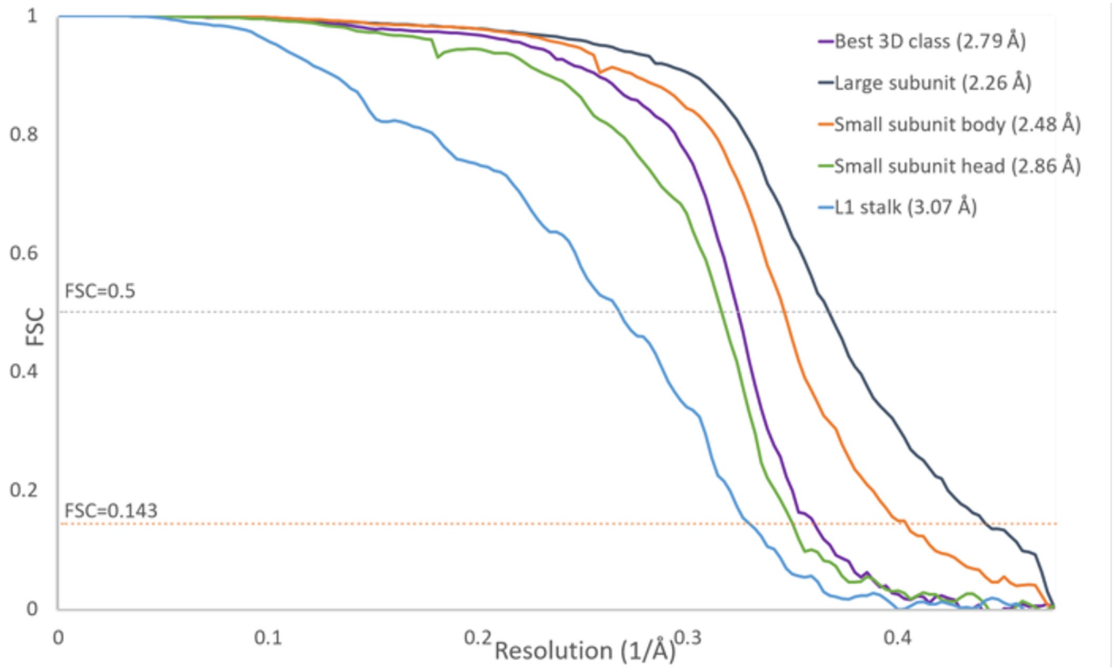

Supplementary Figure 8 – Gold standard Fourier Shell Correlation (FSC) of the single particle data. Purple, best 3D class; dark blue, large subunit; orange, SSU body; green, SSU head; blue, L1 stalk. Indicated resolution values correspond to the FSC 0.143 criterion.

	Chain	Universal nomenclature	Template	Sequence
rRNAs	L50	23S_rRNA	6RM3	AUACCACACACAAGGGAUCGUUUGGCUCCUGGGACGAGGAAGGGCGCAGCAGAGAGCGAUAUGUGUGGGAGCAGC<br>AGCAAUGACCACACGUCCCCGAAUACAGCAUAGUGCUGGAGUACUCCUUGGAAUUAAGCAUAUGAGUAAAGGAAG<br>GAAAAGAAACUAACGAGGAUUCCCGUAGUAGCGGCGAGUGAACAGGGAACAGUCCAACAGUGUAAUUCUCAAAUG<br>AGAAGUUGUCAAAAGGAAGAAGAUGUGAAUCAGAAAGAAAAGCUGAACGAUACAGGGUGAUAGUCCCGUAGUGU<br>CUUCUUACAUACGGUGAGUAGCUGUGCUCGGUAAUGCACAGUGAAGAGGUGGCAGUGUGCAUCUAAGACUAAAU<br>ACAACAGGAGACCGAUAGCAAAGAAGUAGGUUGACCGAAAACAUCUAAGUGAAAUUGUGUGUGGACCCGAAUGGA<br>UCGGGCCCGUCUUGAAACACGGACCAAGGAGUGCACAUACACUGCAAGUUGAGCAGAGGAAGUCUGUGAGGCGUA<br>GUGAAGACGGAAAGCAGUGUGAGUGCGACCCGAUAGACUUGUGAACUAUACCUUGUUGCUGUGAAGGCUGGCGA<br>AAGCCAGCUGGAGGCGGCAAGCCGUAUUGAUCUGCAAAUCAUUGGCGUAAGCAGGGUAUAGGGGCGAAAGACCAA<br>UCGAACAGUCUAGUUGCUGGUUCCCUCCGAAAUGUCUAUCAGGACAGCGCGCACGCAGAGAGAAGCAAGGUAGAG<br>CAUGGGCCGGUAUCCUUAAGGAGACGGACGAACUGCGAAUGUGCUUUUCUUCUAUGCAAGCGUAGUGGGCGAGU<br>UCUGGUAAGCAGGACUGGCGAAGAGGAAUGAACCUGGCACUGUGCUAAGGAACCCAAUGUCUGGACAGAGACCGA<br>AAGAGGUAGGUGAAUAAGGACGGUAGGGCGGUAGCCAUGGAAGUCGGCAUCCGGUAAGAAACGUGUUACAACGU<br>ACCUACGGAAUUCAUCUGCUCUGAAAAUUGAUGGCGCUUACCAGACAUCCGAUGCACAGUUAGCAGGUAGGAGGG<br>CGUGUGUCUACUGGCGAAGGUACCGAGUGAUCGGUGGUGGAGGUAGAUGCAGAGCAGAUCUUGGUGGUAGUAG<br>CGAUAAUUUGUCUUCAGCGGCAAAGACUGAGGAGGAGAAGGGUUUCUUCUCAUAAGAAGAGUGAGCCGGGUCUA<br>AGCGAGUGUGUAGAAGCAUGAGCGAAGGAGAAACAGGUUGAUAUUCCUGUGCCUGUGCUGUCGUACGGCAACGU<br>GUAUUAGCUUGUCGACACGAACAUCUAGACAAAGCAGAAACACCUUUUCUGUUCAAGGGGAAAAAGACCACUGAG<br>ACGGAUCAUCUGGCGACGUGGUUUUAUAUCUGUGACCAGGCCUGAGCCUGGUUUGUUGAGGAUGGUCGUCCAUG<br>AAAAGACAACUAUAUGGCAGCACAGUCCGUACCAACCGUAUCAGGACUCCAAGGUGAAGAGCCUCUAGUGGAUGU<br>UUUACACAUGUAAGGGAAUUCGGCAAAAUGGAUGCGUAACUUCGGGAGAAGUAUUGGUUCAAUUGUGAACUCAU<br>UGUGACAAGGGGAAUCUGACUGUUUAGUAAAAACAUAGCUUCAUGAAGAGAGAUGAAGUGAGUUCUGCCCGGUG<br>CUCGGUCGUGACACGGAGGAAAUGCCAGAAAGCACGGGUUAACGGCGGGAGUAACUAUGACUCUCUUAAGGUAGC<br>CAAACGCCUCGUCAUUUAAAUGGUGACGCGCAUGAAUGGAAUGACGAGAUUCCCACUGUCCCUACAUGUAGACUA<br>GCGAACCCAUUUCCAGGGGAACGGGCCUGGACGGCCAGCGGGGAAAGAAGACCCUGUUGAGCUUGACUCUAGUGU<br>GGCCAAGUUGUGACGAUUGAGGCGAUGUAGGAAGGUGGAGAGCGCAAGCAGAAGUGAAAGACCACUGCGCCAUG<br>AUCAUCACGACACACUUGUUUAAGGACAAAGGCCAGAUGGGGAGUUUGGCUGGGGCGGUACGACCACAGAAUCAG<br>AACGUGGUCGACCGAAGGUACUCACAGCGAGACGAGAACACUCGUGUAGAGCAUAAGGACUAAAGAGUGCCUGAG<br>UGUGAGCACUACUGCUUGCAUGAGGGGAAACCCGGGCCUAGCGAUCCUACGCAUACGAGACACUUGUGGCGUGGG<br>UGUCAGAAAAGUUACCACAGGGAUAACUGGCUUGUGGCCGCCGAGCGUAGAAAGCGACGCGGCUUUUUGAUUCU<br>UCGAUGUCGGCUCUUCCUAGCAUGGCCAUGCAGCCGUGGCGAAGUGUUGGAUUGUUCACCCACUAACAGGGAACG<br>UGAGCUGGGUUUAGACCGUCGUGAGACAGGUUAGUUUUAUCCUACUGUCCAAACAGUUGAAGGGAGUGUGGAU<br>UAGUACGAAAGGAAACCCACACGUGACCUCUGGUGCAGCGGGUGUGCGGAAGUGCAACUGCCAUGCUACGUCUCC<br>UCAAGCAUAGCUGGAAGCCUCUAAGCUAGAAAUGAGUCCUGACUCUGGAAGACGGCCGGGAAGACGACCCGUAGU<br>UAACAGCGGUGUUGUACGAUAUGAGCUUUCUAUUUGUGUAGUAGCGAGUAUUUGAGACACUGGUUGUUACCAC<br>CGUCGUUUAUU
	L70	5S_rRNA	6RM3	AGTTACGGCCATATCTACTGAAAAACACCGGATCCCGTCCGATCTCCGAAGTTAAGCCAATAAGAGCCATGCGAGTATTA<br>AGGTGGGCGACTACTTGAGAAAGCGTGGTGCTGTAGTTT
	S60	16S_rRNA	6RM3	ACCAGGUUGAUUCUGCCUGACGUGGAUGCUAGUCUCAUAGAUUUAGCCAUGCAUGUGUAAGCGAAGCGAUUAGU<br>GAAGCGGCGGAAGGCUCAGUAACGGGCGAGUAUUUAAUCUCUAGAACCGGACAACCUCAGGAAACAGAGGGGAAA<br>ACGGAGUAAGAGCAUGUGUUGUUAGCAUGUGAUGAGAACAACAGUGCAGGAGAGUAAGAAGCCAUCCCAUCAGU<br>UAGUAAGUAGGGUAAGGGCCUAUUUAGACGUAGACGGGUACGGGGGAUUAGGGUUUGAUUCCGGAGAGGGAGC<br>CUGAGAGACGGCUACCAGGUCCAAGGACAGCAGCAGGCGCGAAAAUUACCGAAGCCUCCAAGAGGGCGGUAGUGA<br>AGAGACGUGAAAACAAGUGUGUGUAAAAACCACAUGAGUAACUGGAGGUCAAGGCUGGUGCCAGCAUCCGCGGUA<br>AUACCAGCUCCAGGAGUGUCUAUGAUGAUUGCUGCGAUUAAAAGGUCCGUAGUCGAAGAACAGUACUUGUUUGU<br>AAUGUGGCUUAUCGAGAGCCGAAUAGAACGAGAAGAGUUCAGAGUAAGGAGCGAAGGAGGGCUAGAUUAUUGAG<br>CAGCGAGAGGUGAAAUUUGAUGACCUGCUUAGGAGUAACAGAGGCGAAAGCGCUAGUCAAGAACGAAUCCGAUGA<br>UCAAGGACGUAGGCUGGAGGAUCGAAGACGAUUAGAGACCGUAGUAGUUCCAGCAGUAAACGAUGCCUACGCUGU<br>UGUGGUUAUGAGCACGGCAGAAGAGAAAUCUAGUAGGGCUUUGGGGAGAGUACGCGCGCAAGCGAUAAAUUUAA<br>AGGAAAUUGACGGAAGAACACCACAAGGAGUGGAGUGUGCGGCUUAAUUUGACUCAACGCGGGACAGCUUACCAU<br>ACCCGAUGGCCGUGUGAGCGUAGUACGCGAUAGGUCAGAGAGUGGUGCAUGGCCGUUAACGACGAGUGGGGUGA<br>CCUUUGGGUUAAGUCCGCGAAGUAGUGAGAUCCCUAGGAUACGAAAGUUAUCGACAGGUGUUAAAAACACAGGA<br>AGGAAGGGGCAAGAACAGGUCAGUGAUGCCCUUAGAUGGUAUGGGCUGCACGCGCACUACAGUGGUCACAGCAGA<br>AACAAAUAGAAGUAAAUGUGAUCGAGAGGGAAUGAGCACUGUAAUGUGCACAGGAACGAGGAAUUGCUAGUAAU<br>CGUUAUCUCAUUAAGAAGCGAUGAAUGUGUCCCUGUUCUUUGUACACACCGCCCGUCGUUAUCGAAGAUGGAACC<br>AGGUGCGAACAAGUGAAAGCGAGUGAGUGCAGGGUUCUAGAUCUGAUACAAGUCGUAACAAGGCAGCUGUAGGA<br>GAACCUGUAGCUGGAUCAACUAGUUAAUU

Large Subunit ribosomal proteins	LA0	uL2	6RM3	MGKVIRTLRLQKEHWKRKHHKSKGIVGFPTLKNCEATVEKIVHESGRPAPLAIINYKKEEKNTKGLLVAIEGIYEGQKIQIGENASLELGNVCPIKEIPEGSLVSMVEKKPKDGGKYVKSPGCYATIVYHNKLTNQTTIKLPSGERVRVTGESRALLGIIAGGGKDDKPLLKAVNAYYKYKTKGKPWPRVRGVAMNPVDHPHGGGNHQHVGHPTTVSRNAPVNGRVGLVAARRTGRKKGSRKITVN
	LAA	uL15	6RM3	MFNKKSKSRKLRGHVSHGYGRVGKHRKHPAGRGKSGGLKHLRSLFQRYHPDHFRKLGIVMFHRNKNADFTRTVNVSNLWGLMKLEEQMKFKSSAEVPVVDCRNYGYLKVLGGGDLSVKKPIVVIARQFTKDAEEKINAVGGKCVVAA
	LB0	uL3	6RM3	MSCRNYSAPRHGSLAYCPKKRAKTTKPAIKSFPKDDASKPVHLTGYLAYKVGMTHVVRLSTRRSKTERVLKETQKEVLDAVTILEVPAMRIFGMVGYKSTVNGLKAIKTVYAEHLDEQVLARLRKRNSRSSKLMFKLYPQKYENGDIKRDYELLKRESDVVRVLAHTQVSKIPSLNLKKAHILEIQLNGGTIEEKIEWAIERLEKEIPVSEVFSQQDLIDVIGVTKGKGKEGTTKRFGTTILQHKSRKGKRKVACIGAWHPANVRRTVARAGQLGFHRRTAQNKKIYMMGNGKTPFSTEYDLTSKTINPMGGFLRYGNVKSDFIMVKGGIQGPSKRVLALRKAFAPSNLGKDNEEIVLKFVDASSKKGYGRFQTTEEKKAYFGIKKAEVVEQ
	LC0	uL4	6RM3	MSRQVNIIEQDGTSSRTTSVPAVFNLNVRQDLISFTHKNVAMNSRQPYAVKPGAGMRHSAKSWGTGRAMARVPRVRAGGTRRAGQGANANFCRGGRMFAPTNVNRRWNRKTLLSIRRYASAMAVAATSIPAYVEGRGHRIEQIENIPLVLSNKIDTEKTKEGIKILYNLGLKEELDKVEESKNIRAGKGKWRNRRYVLKKGPLFVYTSETSFTKAIRNIPGVDTERVENLSILDLAPGGQAGRLIIWMEDAFEKLTEIYGEMNEESKFKKGYTMGMDVAENIDVENIFYSDEVQEFIDIPNFIKKDKMVMEKNKLAEIQQYVEIEERI
	LCC	eL30	6RM3	MSRKRSKREESFVDKLPLALKTGKVNLGFKESLKSLVEKKTKLLILTSNFSPIKRKTLEYYAAISNNTPIYYYDGSNNDLSKNCGKYFRIGVISIQDFGEADLMNAMAQA
	LD0	uL18	6RM3	MVSNSINFQSDNKSKKSYFQRFQTKKRRRRLCKTNYRRRTKLIKLDQKNVGEDKSRLVVRITNSKVICQVVKSHLGGDMVVAQATSAELKEFGLKVGFTNYSAAYATGLLCARKILRMEKLDNIYKPKFCTDVDVVEDIEGEKNAYRCYLDIGLSRNSKGAKVFAAMKGASDGGIKIPYSPKIFPGYNKDEEFDQQKLRDRIFGKEVANYMQLLKKEDETKFKKQFSEYIKQNINAEDLEKMYENLFNSIVTKEISKKEKKDYSALKKYKVMKLTKEERDARVQQKLVENN
	LDD	eL31	6RM3	MAFKDDKAIELTIPMGKITIDVSKRWKCRIGIRRLKKFITKTFHDKEAEVQISPDLNKFLWERGMRNVPKRVRVRVNQEPYPKDPSKKVYKLSHVVVSTFKGLGTEAIAE
	LE0	eL6	6RM3	MQSERQQRLNISNIKNYYTADDIPAFAEKLLELHKPAPIELRTDLVQGNVVVVLEGEYASYRVVYLSRTEDNKALCMGLPSINGIGLFEIDERFLLRTSIVLDIRLDKKYRAKESKRSFKKFNTKEKVLTKEENDIEELLLKEIENEKFMKKYFETPYEINNTVDFYEINH
	LEE	eL32	6RM3	MDIVNPSLSKVIDIINKGKSKKQFKRFQSWRVKRVSESWRKPRGIDNRMRRKFGGAPDMPGIGYKKPNAIRFLLPNGYRKIMVACVKDLEALISLNSFYCAEIRHAVGSKKRIEILERAEELGILVINKEGKLVKEEEE
	LF0	uL30	6RM3	MSNVDLIAKKEQYNLRMEELQKKQQEQHLKRVEENKKYAEKRTVELLETYKKIALEQQLSEERMQKENKIYAPKDPHFYAVVLIKSPLRASNKIKKTLELFRLKHINNCVLVKNNESSRQMLQKIRNFVAYGELKLKTLRELIYKRGSCHIFSEKLNITNETLEDAFKNIRCIEEFIYELYFNDERFKELNNFLNPFKLTPPKGGYKGRKAKDIIEGGAVGNHQDKIGELIKKMI
	LFF	eL33	6RM3	MEVIKKIELKERVCAKGTLYSHKRSKKNIYPRYSLITIENVKTREAAMKYVGNAVELFKPESTKEESIHGIIKAVHGRSGAVRAKFSRNISPVEMGSKVYIKLYKMNEDEI
	LG0	eL8	6RM3	MSDIQLTKKQLLSMNPRKLTNKAQMDTRKEVIATRKEALVNAIKLPPALNQFTNYLSEEDLKEVSDILSKFRPETRKERKQRLQNERKGPKPIIVKQGIKHVTKLIEEKKAKFVLIACDVDPIEVVLYLPTLCVKMGIPYALVKTKEDLGKIIKKKPTTCLCLCDVSSDIKSKFENVIAKCNGQFKDNYESAMKVWGSPKEKVKEE
	LGG	eL34	6RM3	MVERQVVRRGSTFNTRSNKRKKVRTPGNRLVFQDVKKKGKGPKCGQCGIKLAGVKEARPAAFSRMRKSCRKVNRILGGVQCAKCVQEKVLDAFLNAEEKRMKELEV
	LH0	uL6	6RM3	MVYVYVEEKVQIPEGCKVEVVNKKVTVTGPKGTVSKDFSHIFVFIDIHEENVRCRLWNCRKQERSKLITCSKLIKNMIDGSMSGYFYELKAAYKHFPITFEIKENGKLLIVKNFLGQKWAREFKMIGDTIIKQGVTKDHLILEGISKEDVSQTAGLIQNNCLPKNLDRRIFQDGVYIATKGLIEEVK
	LHH	uL29	6RM3	MKLLAKDLRNNSIEELEKNLVDAKSEMLQIRQKKQSAILKPNELRNGRRNVAVILTIIREKKYQQLLEKYKDADNIPKELKPRMNRRKRQELTKKQKKISKVGKTKLYTKGNKLIYAFE
	LI0	uL16	6RM3	MGRRPGRCYRYCKNKPYIKSRFCRGVPDPKIRIHDLGNKNAPCVDLPLNVNLISYSKEQLSSESLESARIAVNKYMMVNAGKDNYHVRINVHPYHILRINKMLSCAGADRLQTGMRLAYGKPYGTAARVRFGQRIISIRTKDSNLKMAMEALRRARYKFPGKYEVQKSNEYGFSGIERSQFEELKNNGKLINMGDHVKIIKEKGTVERYVKLCENAIQ
	LII	eL36	6RM3	MKINKLNNASKALSYPVKKLAKTEKAPLTEKQQRSKELKKFAMSIAREVGGLAPYELRATELLRRDEDKRCKKFMKKRLGSLKMAKKKIELLAEEMRA
	LJ0	uL5	6RM3	MAEENPMNKIKIQKLCLNICTGGGEILNKASKVLEQLTGQKPRLSKSRLTIRSFGIRRNEKIACHVTVRGEKAMEILEKGIKIKEYELIDSNFSTQGSFGFGIKEHIDMGIKYEPDVGIFGMNFYVVLEKPGIRVSKRKRVKSRVGNKQRVTKDEAKNWFVKKYEGIVLGK
	LJJ	eL37	6RM3	MSKGTPSNGKKNKRNHTTCCRCGSVSFHKQKGKCSSCAYPEKKMKKMQNRKAMLKKTTGTGKMRHMKKERISRRAGYPGNPILKEIRNKIKS
	LL0	eL13	6RM3	MRGNQMLPNNHFNKGSMKFKNWFNQPMRALKRKEVREQKSKICYPNPLKKLRPVVRCCSMRYNKKVRLGRGFTKMEIEKAGMDIKYAISVGIAVDLRRKNKNEEGLEENVKRLMDYKNKLKFFTTVREARDNKIEQYKGEIMKPVNIIPEVKGIKVEEIATIYGN
	LLL	el39	6RM3	MGSIKTRRIKVQLVSALKKNRCIPQWKRACPGFKQQYNFTKRSWKRKTLKAC
	LM0	eL14	6RM3	KYFSYPLMYFIKPGCLIKKKFSIYTSIVISVIDNNSVVIQSYDKSDNGDVISIDREVINVSKIVPIGNIDIKNKSKKEIDGILVEENRNLKNKDDVLLMNDFERFKEQLKKEVEDMVIEEMA
	LMM	eL40	6RM3	MQLFLKSPNGTSTIDLALENTLENVLTNEGVSTENNRFIYNGKQMDTNRCLFEYGFKGLETIYVTGRICGGKLTDDDKVLAITKFEKQVCRKCYARLPIRATHCRKRACGYSSKLRGKKKRRDESKK
	LN0	eL15	6RM3	MGYLQYLKEIRRDKQSSVMRYLLRVRCWEYRQNTAIHRASGPTYLDKARMLGYRNIPGMCIFRARVRRGARPKKVLKGNTHGKPSKSGVYQRKPAKRLQAFAEIRVGRKCSNMRVFNSYWVGQDRVYKYFEVIMVDPYCDRIKNDKKINWLCKPVMKHREARGLTNGTKTSRGLGKGIRYNQTKGGSKRAAWRNRNTQSLRRYR
	LO0	uL13	6RM3	MIGKRIVIDAIGHVAGKLAGHIAKELMCGIEVVIVHAENVMFTGPIKRGVGKFNSHLNKSCSYNPLRGPFHLRSPSMHLMKVIKRKLPHRKSKGQIALKRLQVFDGCPTEYLSVEKMVCPRDKLEYTTSPIRKSYKLGDLMIKFGWKYESVVEKMNQNEEQFKIEETEKQKVKEQKIEELKKSNKFQQRVEEIMVQMA
	LOO	eL42	6RM3/Alphafold2	MVNIPKTRRTYCIKCAYHTEFKVGLLKKGKDSPRKQGARRYARKQRGVHGQTKPILKRKAKTTKKVILKIECTKCKSSQLKVRGRAKHVEFGAEKKVKGEALIY
	LP0	uL22	6RM3	MAHKYSCDISEATSVRSQYNNIKIKFKNTREVCNIIKTMELNKAIAYLNDVLRFKKCIPVFRFAKKCGRTPQAKEFGTDKGKWPVKSVAFVKKMLEDLLVEAEKKGLGTSELVIKHIQVNQAPKIYGRKHGAFGRIKPYNKSPSHIEVIAVKREHNIKEGDVVEVKE
	LPP	eL43	6RM3	MSRKTKKVGITGKYGSRYGSSLKRRAMICMNAQSKRYCCSFCGKTTVKREVIGIWSCRFCGKKVSGGAYVPITNQQVEYKQVINRMKME
	LQ0	eL18	6RM3	MLKQKMFEHYKKKAILFDESRVPRTKNVFYQSLFSLYKKVCENTENEIVHKITKRLTMSRTERHPLKLSNIIEQANGKEIVVFVGKVLDDDKILEIPKITIVALKISKQAKEKLTLNGGEFYTLDKLFYISKDLKDVALVKGDKNNRKCTRYFGASGDKGSETYPRTRKGARRGERRIKNKKK
	LR0	eL19	6RM3	MKLPRVKELSADILKCGVRRIYLDTFEPSKLSAATSRDDIRKLIKDGLIVRKAQKIHSRYHANKLMREKEKGRHSGPGKVKGSKNARMPEKDKWIKRIRDLRNTLKELRDKGEITKTEHKMYYQKTKGNGYKNSAALMGAIKQKHDDERRMKEIEEQAKALKIAASEK
	LS0	eL20	6RM3	MAINEYKVYGIKIDDNGVVGDEVYAHIVFATNELLAKSSFNKILKKQKKIKPKNTKVIKVEEIKEDNKDLDVKNYGIDYYYKSKRCDHKGYKEFRGVSRCHAVDKLLCDIGGRHSVKRNLIFIMKVKELTVDEIKKDKIKIFAMEDVEYPAYRNVLRSNKLFVSENEKLFN
	LT0	eL21	6RM3	MVSSHGYRRQTRNLFSQAFRKHGMPNTSTYLTQYAVGEYVDIKVNPAIHKGMPHKYYHGRTGIIKEVNRGSVIVLFNRILANKTVERKVIVGIEHIRKSKCQDEYLKRLEINKKIIEEAEKKGEVPVLFKRQPKGPKPAVEMLMNNIIELSYDKVESQ
	LU0	eL22	6RM3	MSEAAVQDQSITYTLDCTKCTEQSLFSTDDLKEYLLGNIKYNGKKGQLGKKVLLSNTGHAVDVFFAKKTSKRYLKYLIKKFLRAKGLSDWVRPIAGDKQSYKFSFFSKQAGEE
	LV0	uL14	6RM3	MPGKGKQPLDAIKNRYGFTRGIQVGTVLACADNSGAKMLKTIGVKGVSGRLNRLPAAGPGDVIVASVVKGRSDMRKKIVLAVIIRQKQMIRRKDGVRLQFEESAAVIISNKGDLKGTQISGPVLREVCDIWPKISSQAGSIY
	LW0	eL24	6RM3	MFYEGTCVYSGYPVPRGKGTYFVSLDDKSSLFFSSKYKNFSDKKIKAKRVKQTLMSKQFCGKVESVHTEKKKKIVVQKIVRGFPALPKDAVEKIREQNKDYKKKIVDEKKVQHGKKVGKIEQKQMKQKQKY
	LX0	uL23	6RM3	MPKLNRHKASRLDKKIKQKKLIEKPLGFSSLDIIKYGIGHEGAAKLIEEAGTLTFVVDQRADKINVKKAFEEIYGEKVKKVNINNTMKGVKKAYIRLVELGNAAVVATKAGIL
	LY0	uL24	6RM3	MKFNKNVTASRRKNRKAHFTSIGLERTKLMNAPLSKDLKEEYGIKRIAVRPGDMVKVVSGEFKKKEGVVMKVNRHDIKIEVEGCFVQKEDGKKINKGIHPSNLRIIKLSMEKDRAEIINKKVEAMKSEVKA
	LZ0	eL27	6RM3	MKPTFIIRINLFFPFKKYFFLFHIFFLFFIFFCPMSETLFEKNMFVLLNRGRFAGNKGIIVDTDLENNRIIIAGVSKIPKNKKRKTIVFLKKMNPMHIMATTYKRDIGLSLDNLDSIFEDKVKKAVALNRVREIFNKNKNNEGFAWLYKKLEI

	Chain	Protein	Template	Sequence
Small Subunit ribosomal proteins	SA0	uS2	6RM3	MAVRVVPLSDKVAKAMVQCCCHLGGVTSSTSLKEYIFGKREEDKVNIIDIKKMWEKLNLAARMICSIPNPKDIVVVSSKDFGRKAVIKFAESIGATAVTGRFIPGSFSNYEIKGVREPRLVLVSDCFADKQVVKEASYVNAPCIAFCNTDNDIEFVDMVIPMNNRSQSAIGAGFCILSKLVNYMKGNIDKVEDKLRDHIELYTYRDSDELEQLYQEQKIEEEADMVLKDESDE
	SAA	eS26	6RM3	MTKKRSNNGRSKKNRGHTRCVRCEHCSAMVPNDKAIKKFVVRSVIDAASLDDLKIATIYEEYEVPKSYYKLNYCVSCAVHIKLVRVRSRWERKIRKEQRVSA
	SB0	eS1	6RM3	MAIKADNPKMRKGMKKGGKKKVEKDSFLKKEKYNLRIENFPVREVGATLVNKQSTVAALEKKLVGRVFEANQGDITTSDSANCYRKFSFKIDSLAGTNAHAIFNGMCLTSEKIKGMVKRGLTLIEGVKDVVTKEGITLRILTIAQSKKEKNSTKKTAYAKTSVVKEVRKNIFRIVDNELNGKDIEQIVKKLQMETVGKEIEKECQKIIPLQNCHVLKVKVVKRPVKEEVE
	SBB	eS27	6RM3	MIAKNLLYPTVEEIKKNNKRKQLFQEVKGYFMEVKCAGCATLNVCYSHSQKTISCKGCSMVILRSTGGKAKISNECSYMVIS
	SC0	uS5	6RM3	MNSEETTKTSSQEQRRPKRAPREEAEWFPKTSLGKLVKAGRITLDEIFGHSLCIREPEIIDYFLKATLKEEVLVIKSVQKQTKAGQKTRMKVVLALGDHNNYIGIGSGVARDVVSAINAAKVVAKCSMRPIKKGFWGNEIGEAHTVPVKALGKCGSVKVQVTPAPKGTGLVAGGVSKTIFKLAGIKDIFTSSKGQTCTTENFARASLYAITNSTNFNMPNVIEEKVVELNPILKNAEFLIEQEQRDLR
	SCC	eS28	6RM3	MSEEVYIGEVLQTLEKTGPGGGLTMCQVQLKDQKRTLYRAIMGPIAVGDLVALLDCEREHRRGRF
	SD0	uS3	6RM3	MQNKSIFPQLIFFNIFFQIIFFTLMFNPEASSIKNRFVERGCYYAEINEFLSKLLQKEGFSGLSLRLHERPIRVVIRLEKTTEVLKDNSVLLKQIKALIGKRLQEDVSNIDILIEMVKNKGLCPAIQAEFIRSKFEENIPYRRSVVSAIKNIKSAGGQGCVIEVSGKLKGQRARSVKTIDGKIIHAGQPKEEFMRYATTSVLLKQGIIGIKVGIMLPQDDEGIKGPVYDMVDKIKVLPIKEE
	SDD	uS14	6RM3	MNKQMIKYNADLETTFTVERKDFGRTNRRCRACFAYNGVIRKYDLMICRRCFREYAGDIGFKIYD
	SE0	eS4	6RM3	MVRGPRKHLKRLNAPKSWSLDKNGGVFAPRTSTGPHSMQESIPLCLLLTRILKVASNNKEIKHIMKNRLITVNGNIRTDSRYPVGIMDVLSIKKTNEHYRLLYNIAGKFVLHKITEEEAQYRIAKVTNKKVVKGNIPHTYTSCGGSFKYADPSISIGDSVKIDIKTSKIVENSSLEVGKVVYLIKGKNIGCLGVITGIEKREGTHDIVNIVDKVGRNFSTIFSNILVVGADDNPWVTFTKDEGIKYSEYEQSIKEYGPMNEEKEEESIPEVVETSEVETRHPTRARG
	SEE	eS30	6RM3	MAKAISLNKAGKVRGQTPKVEKSEKEKALTGRAAWRAKFKRRQELFTGVGKFKFNKQGDF
	SF0	uS7	6RM3	MRYTETSGDIKLFGRYPYTDLEVKDITLEKYINVASKVILPHTSSRLNTKKFGRTKIPVVERFVNAMMRKGRNNGKKTLAMRIVKDAFFIIEGVTGQNPIQILVDAICNSGPREDSARIGKGGVMKRSSVDVSPLRRINIAIFLLAKGVRNSTFKDIKMLPEIIAEELINAAKNTQSSFAVKKKEEIERIAKSNR
	SFF	eS31	Alphafold2	MQIFVKTLTGKTITLEVESTDSIENVKAKIQDKEGIPPDQQRLIFAGKQLEDGRTLSDYNIQKESTLHLVLRLRGGKKKKKKKSYTTPKKVKKPKQDIKQIILTQFSVNKDGECKPLRNSCSTCGPSVFLGRSGNKEYCGRCYTVAILNK
	SG0	eS6	6RM3	MKLNISYPTNGTHKTFKLDFKAEQRLYGKKTLDTFDGSIIKPEWEGLQFQITGGSDVQGFPMIKTLLTDKRKKLLLKKGDKGFKPVNRKGLRKRKTVRGSIIASDISMVCVSLIDAKEVAIEGLTDVVVGATHWPKRFNKLKAKAGFSEDADVTAEQVLKVIKDAILEVNGGDIKKLPKLKVSRYQTEKQKERKAEKIKLIEERKKKSEKERADFLKKYPDWQKKFATKA
	SGG	RACK1	6RM3	MISLNEKGSQYVHKQMVTCLEVPNEGNSDILYTASRDKKIYSILLKPEEDKVGKYIKSYEGHSGFVNCITTNSSNDKIISGSSDNTARIFDVESQKSVVLKGHTRDITSISINTEDNKIITGSVDGKIMLWNTQGKCAEVFDGECQNTHTSWINCVAFRPFNSNIVVSGSEDGTLKIWDIEEKRVMETFINGFSVTEIKEKGIEIERKSAVTSLSMSPDGSLCAYGSKNSMVYVFKFDDNKCVINFDAGSTVKALAFGLTDVSIACATESEIILWDILKNVRIARIDYSSYGNGVYCTSLVWAKDMLLAGFSDGRITAYERTIIEE
	SH0	eS7	6RM3	MSQSQSEMQVAEVIKKVCGSVIKDSLQSALKTLRIKLISDSDGSNIMVITLPLPLLDAIQQYFSEIQNKLQKQFSEFQIFFIREAVYNPNNTLNKKLQENWLKDLCFPSVVNQRMTVVKEGGNIKEEKVFVERKSEFTEEEFYSMKLVFKELTGRDVEYGLRYY
	SI0	eS8	6RM3	MGISRSNRHKRKETGGKKGLHEKKRKNNCGRPPSNTRIGERRVKQVRVRGGNIKHRALRLDTGNIACKTHNFTLNTSIQNVMYHPSNNEFMRTNILTKSAVVKINGQPFKHELEKDQQFESKDPVLFDNLRKGNCYAIITSRPGQVGMADGYLLQGEELKFYMEKFKKKSVKV
	SJ0	uS4	6RM3	MKFFSTLMVKSKGIKKIVVPHRPFERERLVSEMYIVGKYGLRNKRELWTIQKMCDSFKLRAKDLLITPDEKEFTLSSRRLLKKLAEWGVFMEDVDYNNKQSLTNNLEKLLDLAPTSFLERRLQHRIFDLGLASSVHQARAFIHARRVKINGSVVNKPGYLVKDGEDGFIEVVESKRSALVAAEE
	SK0	eS10	6RM3	MQLSREDQVAIRKYLFRESICLVKDKDNGNHPELNINNKQVMKFLRSYVSRGLVEKVFVWQHAYFLLKSEGVDVLREELYLDETEVPLTYMRETYEAPKAEVGKVQE
	SL0	uS17	6RM3	MLNEISVSKVFPKQAGVFNNPYEKDSKRRVKDVSEIYKAPESARNSTYIDKNCPFTGEIKIKNNFMKGEVLKMKQPTTIVVVKRYLHYVKKYKRYERRYKNVSVHLSPCFKGLVNVGDTVICGETRPLSKTKRFVVVGFEKKEIKEKKLKMLSEI
	SM0	eS12	6RM3	MSEIVVPMGSEELTLDQALYQVCRISNANNRMVKGIRQVSKKLLDSNLKLVLLSNEIDERSANIIISLAKKTNIPLIKVDDSITLGRYAGFEKRTISDEIKVAKCGCAGIVDYVQPSQGQVFLMNAIKQG
	SN0	uS15	6RM3	MARMYSNGKGQSKSVTPYNLTYPTYIKHSKEEIVDVILNLARKGTPPSQIGNILRDNYGVGKSFAVTKTKILTLLKIAGLKPVIPEDLNNIVKKCVNIKAHLAKYTNDKSSKYQLIQMQSCLYRVARYYKSKNELPAGWKPPF
	SO0	uS11	6RM3	MSEPMVEQRLNFALLFIKATKNDTIIHATDLTGAETLAKSSGGSKVKAHRDERDPYAAMLATQDVAAVLKEKGYNAVHIKIRGKGGNQSKIIGPGGSTVIRTMMRSEFKLGRVEDVTPVATDSTRKKGGRRGRRL
	SP0	uS19	6RM3	MAKKQNFQKKKKSVAVESMGDASARSKRTFKEFSYKGVNLETLTSMSISEFKELLPSKLRRSINRGFSAAERDFIKKCIKARSENTGLERPEQINTNTRNLPILPTMIGLQIGVYSGRDFMPMVIKEEMLGRRLRDYVETRKLCTHGRPGVGATSSSKFVPLK
	SQ0	uS9	6RM3	MSIAQVITKGTRKTAISKCVCSESNEFQLMVDNMTLDVHHDNFLVAKIKEIMKIIGYEHLENLKFELTTRGGGPISRAYAIRQAFAKAVISYFGKYYDEQRKQDIKEQIMKFDRAALVADPRRVEPKKYGGPAARAKYTKSYR
	SR0	eS17	6RM3	MGQIKGKSVRTASKVIVERYFNRLTNDFYDNRNVVIDVAEIRSKKLRNKVSGCVTKLYKRVKKNGVPGLYIKEHEEEREKKESFIPKISILDEDKVEVDEVTMEMINQYEINGDYVLAKK
	SS0	uS13	6RM3	MQAKESKLVFNNNPLPIQAMIRMFNTNIDGKKQIIKALTTIRGIGDRLSLAILRRAKIPTDKRAGELTEDELNEINEVILNPQKYDIPEWMLNHQNDPIDGTTKHLIGNQIEGTIRLWNERGKKIGLVRAFRTSLGLKVRGQRTKSNGRGGRTMGVSRKK
	ST0	eS19	6RM3	MNTEIFKVRHDTFIPALSEYLRTNKTVVPIKNFDIIKTSRGKETSPLDINWYYTRAASIIRKLLISKAKNEKPLSIVRFSSEYGCAKDRGNRPNKHVRASKGIIIKILNDLQEKGWVEKKENGWTLKKEAEEVSKQLIEKLIN
	SU0	uS10	6RM3	MNDVEKKYDIETPVPEKLINVELRLLCSNKKLLSDLSNRYYNYLFQNSPTAKKPRVLQPFICDVTTRKSPCGQGTATWSKYKMIIHGRMFALQMTHQMLKDSAAFLQNTDVEVRLTLKG
	SV0	eS21	6RM3	MNSFQRVCDFSLKPISSQDRSSVQINMVQLDERGRATKDLMIFNVSGTVRKNGKIDSMLYERINEME
	SW0	uS8	6RM3	MMDKLANLCITINNANRAQKRSVVIRRISKVCRLFLESMQKHGYITDIVYIDDHREGKAVVGLSGRLNRCGAICPRYDVELKDVERYRANLLPSRGFGHLIVTSSKGLIDHRECIQHNTGGKILGYFY
	SX0	uS12	6RM3	MYGLLAARNLKKNRHKRRYADKDFAKKARGDIYQNAVGYSAPHAKGIVTEKLGVEAKQPNSAIRKCVRVHIVRLNKKVIAYVPHDGGLNQIEINDEVIVEGLGKKGCRSKGDIPGVRFRVCKVQKVSLKALVTKKKERPAR
	SY0	eS24	6RM3	MVSSFSNFQSFSIEILSRYDNELLHRSEVDLNISHENAMTPSRSDVAQKLAQLFNTGVNNIVIRGTKTFFGSGNSKVKVKVYNNMDQLKKIEHFCELKRIAEKSKTEMQLVEKLPRRTRKDNRKKRNKMWGTEKRRMKKAEKKNSR
	SZ0	eS25	6RM3	MAKKVLESKTSKAEKISNSTKSKKKWSSGKVAEAVKRHERVEDDIFNKIVKDVKNMRILTRTTLSERYNLSLNISIKLLRFLEENQIIKLISSHRGSKIYAGSKYEPLKVVEQPKEVEATVDNDQEWA

Supplementary Figure 9 - Molecular model of the *S. lophii* 70 S ribosome.

Individual rRNA and protein chain sequences are listed, along with their ribosomal universal nomenclature and the templates used for model building. Protein sequences modelled in the map are underlined.

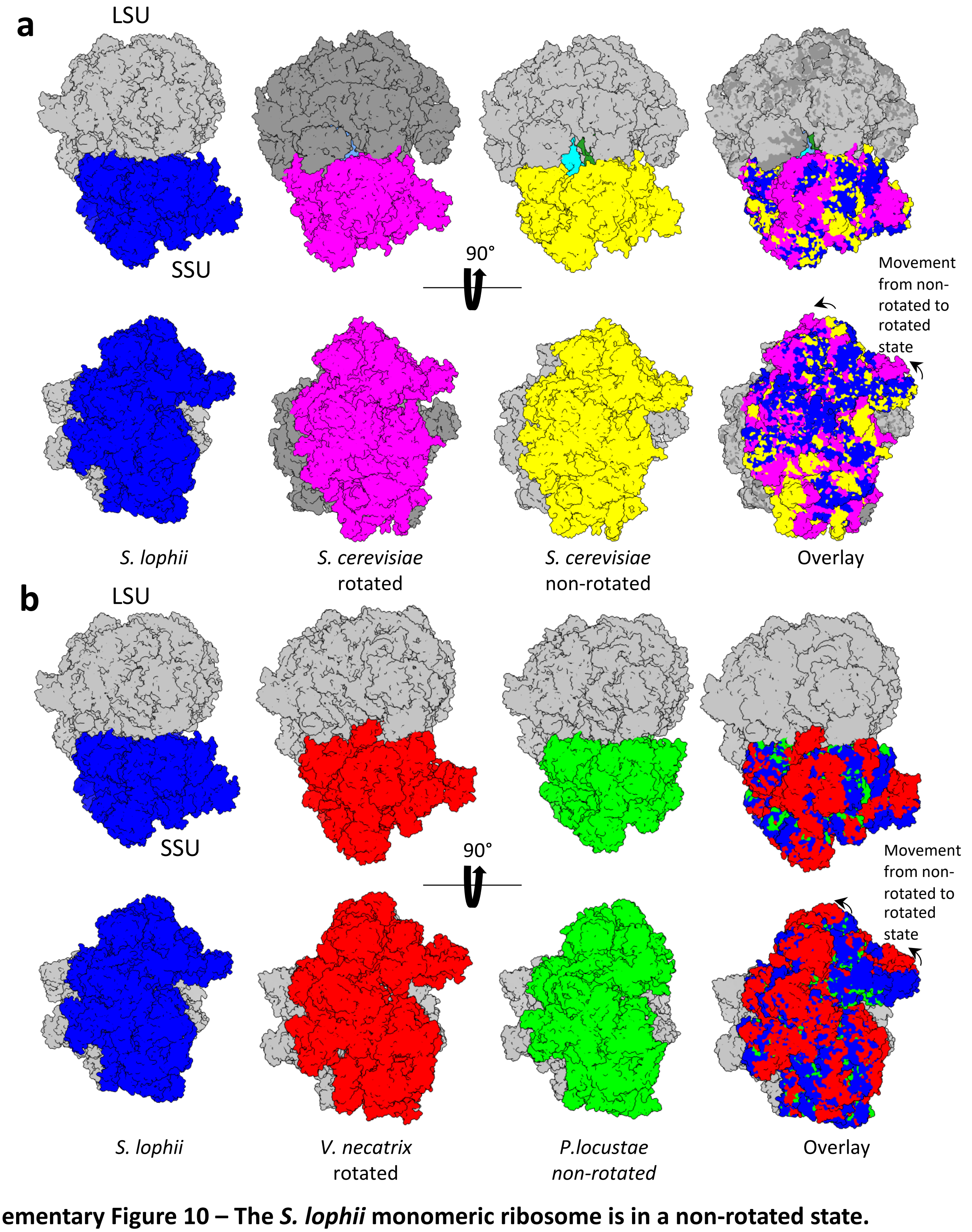

Supplementary Figure 10 – The *S. lophii* monomeric ribosome is in a non-rotated state.

a Ribosome structures (left to right: *S. lophii* (7QCA), *S. cerevisiae* (3J77; rotated state), *S. cerevisiae* (3J78; non-rotated state) and all structures overlaid) shown in surface representation. Ribosomes are displayed in a side view in the upper panel, and then rotated by 90 to show a bottom view in the lower panel. Large subunits are coloured grey and small subunits blue (*S. lophii*), magenta (*S. cerevisiae*, rotated state) and yellow (*S. cerevisiae*, non-rotated state). tRNA molecules bound to the yeast non-rotated state are coloured in cyan and green. **b** Microsporidian ribosome structures (left to right: *S. lophii* (7QCA), *V. necatrix* (6RM3; rotated state), *P. locustae* (7ZU5; non-rotated state) and all structures overlaid) shown in surface representation. Ribosomes are displayed in a side view in the upper panel, and then rotated by 90° to show a bottom view in the lower panel. Large subunits are coloured grey and small subunits blue (*S. lophii*), red (*V. necatrix*, rotated state) and green (*P. locustae*, non-rotated state).

a

```
Vn MDF1     1   MKYELIAKADFDNIKGLSCDQTYPAILSCTACSFVHPKVVILTSESTIKEPNVYKCNWEM   60
Sl MDF1     1   MKYNVYIEGTFSGIQSIMVEQVYPITIKCSGCNLVHKKDVILSIDSINYTDKDEKVNLKV   60
                ***::  :. *..*:.:  :*.**  :.*:.*.:** * ***: :*      :  * * ::

Vn MDF1    61   KCHSCKNDIKVSIYKSPSLSTVNIKDRYEDDVLVSYNPVNKNECLLSILECSGGEIREIK 120
Sl MDF1    61   KCASCTRQMYILI---KSMAT----ELYEEKFYCAKK--EDNRYLASQLQGRGCYIEKIK 111
                ** **..:: : *    *::*    : **:..   : :  :.*. * * *:  *  *.:**

Vn MDF1   121   NVPLNILTDDLRLFNEKVVDEKRSLAEVYGNNKTYSIINYELEIRRVK 168
Sl MDF1   112   DANIHVLSEKGRIYPDIVFDEGYWREEDNMNN-TSSIEDFNIVLESVK 158
                :. :::*::. *:: : *.**     *    ** * ** :::: :. **
```

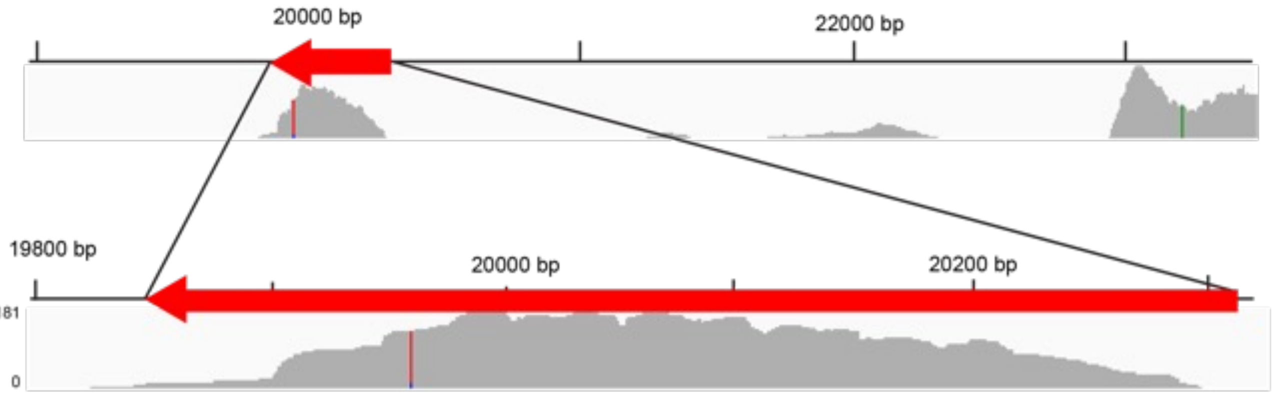

b

```
Pl Lso2     1 MSRTRNAQKKYEGMMRKLKAKEERMRKEEELKEEEAKKFWSIGAKDESKKQALELKRQEK 60
Sl Lso2     1 MSRTRNYVKKKEGRLRKLTKLEEAKRLEEEKKEEELKEYWSVGAKDYSEEMAKEEKRLAK 60
              ******  ** ** :***.  **  * *** **** *::**:**** *:: * * **  *

Pl Lso2    61 IARRAMRKKIYEEELGL------- 77
Sl Lso2    61 IEARKQRME--QERMEMEEIEKMK 82
              *  *  * :  :*.: :
```

0

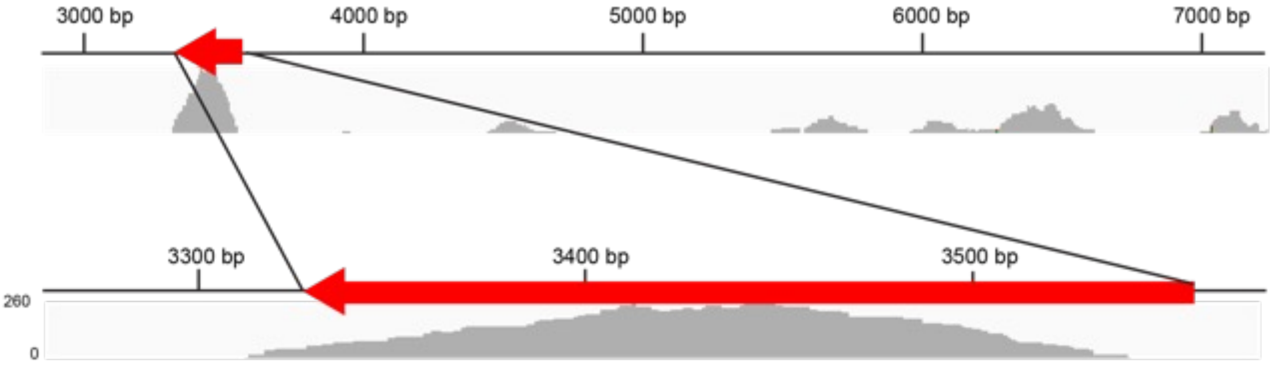

Supplementary Figure 11 – MDF1 and Lso2 homologs are encoded in the genome of *S. lophii.*
Sequence alignments of ribosomal hibernation factors. **a** BLASTP search of the *S. lophii* protein database with the MDF1 protein sequence from *V. necatrix* resulted in a hit for hypothetical protein SLOPH_1662 (EPR78075.1) with 29% sequence identity and 51% sequence similarity. **b** A putative Lso2 protein was identified in the *S. lophii* genome on contig ATCN01000495.1 using TBLASTN with the Lso2 from *P. locustae*. The sequences have 52% identity and 68% similarity. RNASeq reads from the *Spraguea lophii* transcriptome (SRX312311) were mapped onto the Celtic Deep *Spraguea lophii* assembly (GCA_001887945.1) using Bowtie2[51] with default settings. Upper contigs show the context of the genes of interest and RNAseq coverage compared to immediately adjacent regions of the genome and lower contig regions focus on RNAseq coverage of the gene of interest.

Preliminary annotation	Protein name	Number of peptides	Peak area
60 S ribosomal protein L10	uL16	14	5.51E09
40 S ribosomal protein S4	eS4	29	4.90E09
60 S ribosomal protein L7	uL30	30	4.69E09
40 S ribosomal protein S0	uS2	24	4.38E09
40 S ribosomal protein S2	uS5	21	4.14E09
40 S ribosomal protein S3	uS3	21	4.08E09
Ribosomal protein L23	uL14	11	4.02E09
60 S ribosomal protein L4	uL4	30	3.85E09
60 S ribosomal protein L3	uL3	39	3.76E09
60 S ribosomal protein L12	uL11	8	3.71E09
60 S ribosomal protein L17	uL22	20	3.62E09
60 S ribosomal protein L10a	uL10	19	3.61E09
40 S ribosomal protein S14	uS11	11	3.52E09
60 S ribosomal protein L18	eL18	23	3.47E09
60 S ribosomal protein L5	uL18	26	3.29E09
40 S ribosomal protein S20	uS10	13	3.26E09
60 S ribosomal protein L8	eL8	14	3.05E09
Ribosomal protein L15	eL15	8	2.91E09
Ribosomal protein S19	eS19	17	2.90E09
60 S ribosomal protein L11	uL5	9	2.80E09
Ribosomal protein L29	uL29	8	2.73E09
40 S ribosomal protein S12	eS12	13	2.65E09
60 S ribosomal protein L6	eL6	7	2.62E09
60 S ribosomal protein L21	eL21	20	2.57E09
60 S ribosomal protein L20	eL20	18	2.55E09
S7XVN9 uncharacterised protein	-	10	2.50E09
60 S ribosomal protein L23a	uL14	10	2.45E09
40 S ribosomal protein S9	uS4	19	2.44E09
40 S ribosomal protein S7	eS7	12	2.43E09
40 S ribosomal protein S11	uS17	23	2.30E09
60 S ribosomal protein L26	uL24	14	2.30E09
60 S ribosomal protein L37a	eL43	6	2.30E09
60 S ribosomal protein L9	uL6	17	2.25E09
Guanine nucleotide binding protein β	RACK1	25	2.21E09
Ribosomal protein L34	eL34	6	2.17E09
40 S ribosomal protein S16	uS9	7	2.15E09
60 S ribosomal protein L8	eL8	19	2.11E09
40 S ribosomal protein S26	eS26	4	2.08E09

Preliminary annotation	Protein name	Number of peptides	Peak area
40 S ribosomal protein S5	uS7	17	2.06E09
40 S ribosomal protein S15A	uS19	13	2.03E09
60 S ribosomal protein L32	eL32	17	2.00E09
60 S ribosomal protein L27	eL27	11	1.94E09
40 S ribosomal protein S13	uS15	13	1.90E09
Ribosomal protein L13A	eL13	18	1.84E09
60 S ribosomal protein L35a	uL29	7	1.80E09
60 S ribosomal protein L31	eL31	11	1.76E09
60 S ribosomal protein L36	eL36	10	1.74E09
40 S ribosomal protein S25	eS25	8	1.70E09
60 S ribosomal protein L19	eL19	14	1.64E09
40 S ribosomal protein S24	eS24	16	1.62E09
Ubiquitin/40 S ribosomal prot S27a	eS31	9	1.61E09
60 S ribosomal protein L13	eL13	21	1.51E09
40 S ribosomal protein S8	eS8	11	1.51E09
40 S ribosomal protein S10	eS10	12	1.39E09
40 S ribosomal protein S6	eS6	13	1.33E09
60 S ribosomal protein L39	eL39	9	1.23E09
S7W9F9 uncharacterised protein	-	9	1.20E09
40 S ribosomal protein S29	uS14	8	1.10E09
60 S ribosomal protein L22	eL22	10	8.56E08
40 S ribosomal protein S18	uS13	8	8.50E08
60 S ribosomal protein L3	uL3	12	8.40E08
S7XRH7 uncharacterised protein	-	1	7.91E08
S7XL41 Uncharacterised protein	-	8	6.31E08
Ribosomal protein S21E	eS21	7	5.89E08
Ribosomal protein L24E	eL24	8	2.79E08
60 S ribosomal protein L44	eL42	6	2.52E08
**S7W5K5 uncharacterised protein**	**MDF1**	**12**	**1.75E08**
S7W996 uncharacterised protein	-	13	1.72E08
S7W9A1 uncharacterised protein	-	7	88280278
S7WEE5 uncharacterised protein	-	8	73470297
S7XUM1 uncharacterised protein	-	7	61605781
S7WA57 uncharacterised protein	-	2	51516554
60 S ribosomal protein P0	uL10	8	46350286
S7XJY0 uncharacterised protein	-	3	39028496
S7XJ25 uncharacterised protein	-	7	24881672

Preliminary annotation	Protein name	Number of peptides	Peak area
S7W9S4 uncharacterised protein	-	5	24124621
S7W6A0 uncharacterised protein	-	1	22330513
S7W535 uncharacterised protein	-	7	18090219
60 S ribosomal protein P2	P1/P2	3	16879465
Ubiquitin-40 S ribosomal prot S27a	eS31	4	16446609
S7W9Z4 uncharacterised protein	-	2	9578404
S7W8B4 uncharacterised protein	-	6	8240642
S7XIT1 uncharacterised protein	-	3	6803198
S7W633 uncharacterised protein	-	3	5718484
S7W7U9 uncharacterised protein	-	3	4781359
S7W9D1 uncharacterised protein	-	1	4668589
S7XF67 uncharacterised protein	-	1	3290196
60 S ribosomal protein	-	1	2760956
S7XUL4 uncharacterised protein	-	2	2582596
S7W4Q0 uncharacterised protein	-	2	2471210
S7XKV6 uncharacterised protein	-	1	1905871
S7W5D5 uncharacterised protein	-	1	1427322
Ubiquitin-60 S ribosomal prot L40	eL40	4	1401421
S7XLB7 uncharacterised protein	-	1	1030522
S7W9X9 uncharacterised protein	-	1	927439
S7XJ96 uncharacterised protein	-	1	-
S7XH02 uncharacterised protein	-	1	-

Supplementary Figure 12 – Mass spectrometry analysis of the purified *S. lophii* ribosome sample. List of ribosomal and uncharacterised proteins identified in the mass spectrometry analysis of main ribosomal peak (point 2 in Fig. S6a). The proteins are presented in peak height order.

a <u>Single particle map and model (EMD-13892 / PDB-7QCA)</u>
Masked: 2.4 Å
Unmasked: 2.4 Å

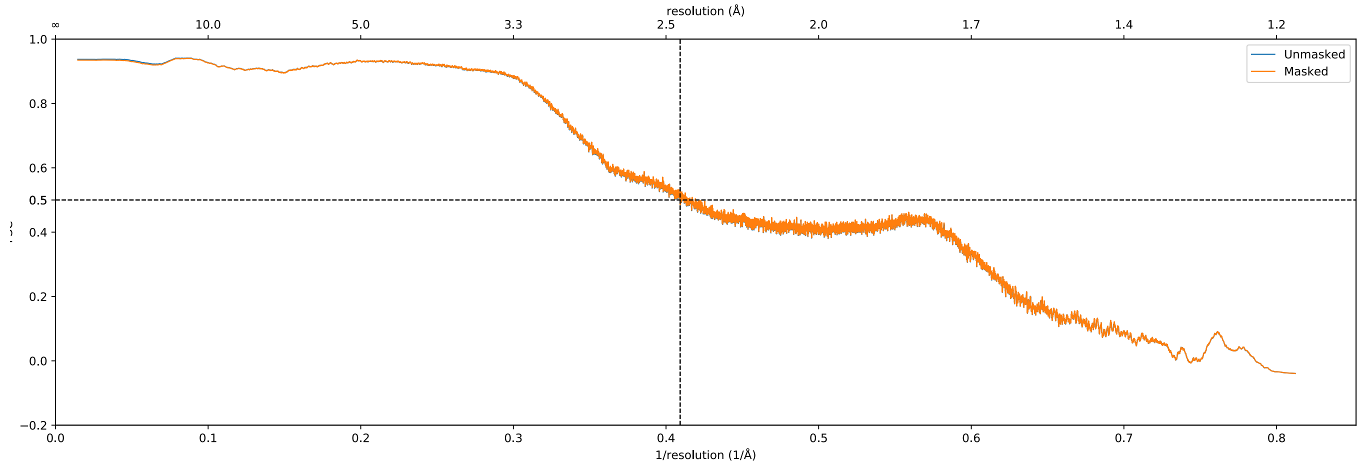

b <u>Sub-tomogram average map of the half dimer and model (EMD-17448 / PDB-8P5D)</u>
Masked: 11.4 Å
Unmasked: 11.6 Å

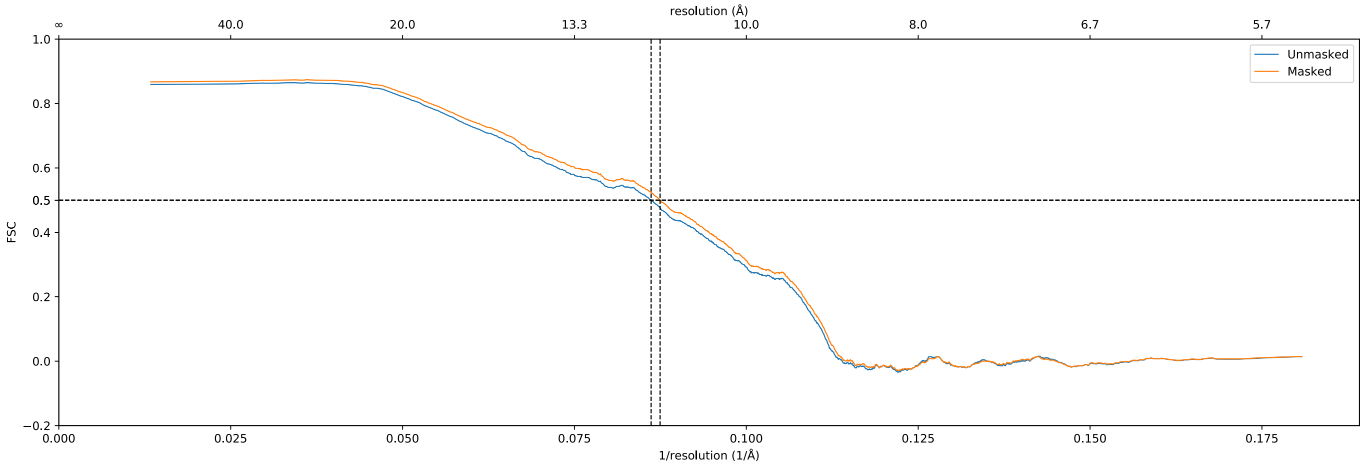

c <u>Sub-tomogram average map of the full dimer and model (EMD-17457/ PDB-8P60)</u>
Masked: 18.2 Å
Unmasked: 18.5 Å

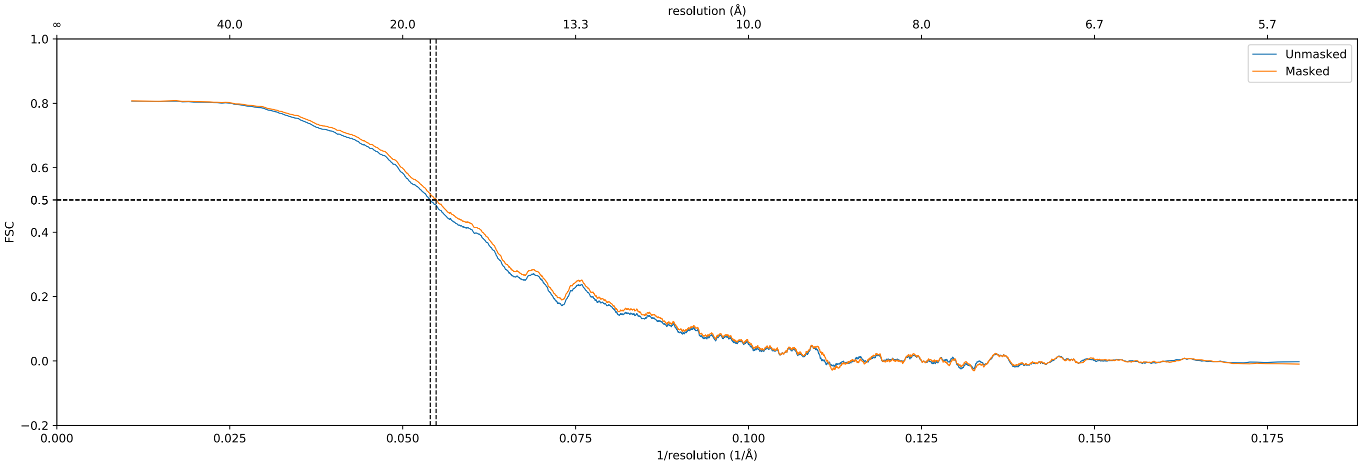

Supplementary Figure 13 – Map/model FSCs.

a Map/model FSC for the single particle map. **b** Map/model FSC for the half-dimer subtomogram average. **c** Map/model FSC for the full dimer subtomogram average.

**Data Collection**	**Dataset 1**	**Dataset 2**
Acceleration voltage (kV)	300	300
Pixel size (Å)	4.377	4.53 (2.265 super res.)
Defocus range (µm)	-4 to -6 (0.5 step)	-2.5 to -4.5 (0.5 step)
Frames per tilt	10	26
Dose per tilt ($e^-/Å^2$)	1.90	1.97
Tilt range (°)	-60 to +60	-60 to +60
Tilt step (°)	2	2
Total dose ($e^-/Å^2$)	116	120

**Depositions**	
**Dimer**	
PDB ID	8P60
EMDB accession number	17457
Resolution (Å) (Masked FSC = 0.143 / 0.5)	14.3 / 20.0
Resolution (Å) (map/model; FSC = 0.5)	18.2
**Half-dimer**	
PDB ID	8P5D
EMDB accession number	17448
Resolution (Å) (Masked FSC = 0.143 / 0.5)	10.8 / 15.5
Resolution (Å) (map/model; FSC = 0.5)	11.4

Supplementary Table 1 – Electron cryo-tomography data collection and subtomogram averaging statistics

	Dataset 1	Dataset 2	Dataset 3	Dataset 4
Acceleration voltage (kV)	200	200	200	300
Pixel size (Å)	1.054	1.054 (0.525)	1.054 (0.525)	1.06 (0.53)
Defocus range (µm)	-1.2 to -2.5	-1.2 to -2.5	-1.2 to -2.5	-1.2 to –2.5
Frames per movie	40	40	40	40
Total dose (e^-/Å^2)	40	42	41.34	41.6
Number of micrographs	513	1816	9192	15 989
Number of particles	30 743	83 575	206 256	657 305
Particles in final refinement	14 949	42 390	114 880	113 721

	**4 datasets combined**				
**Maps**	**Overall**	**LSU focused**	**SSU head focused**	**SSU body focused**	**L1 stalk focused**
Resolution (Å) (0.143 FSC / 0.5 FSC)	2.79 / 3.09	2.26 / 2.73	2.86 / 3.16	2.48 / 2.89	3.07 / 3.77
EMDB accession number	All submitted under EMDB-13892				

**Model Refinement – Overall map**	
PDB ID	7QCA
EMDB accession number	13892
Resolution (Masked FSC = 0.143) (Å)	2.79
Resolution map / model (FSC=0.5)	2.4
**Model Composition**	
Non-hydrogen atoms	170,634
Protein residues	10,629
RNA bases	3,972
Ligands	368
**RMS Deviations**	
Bond length (Å)	0.009
Bond Angle (°)	1.609
**Validation**	
Clash score	2.0
Rotamer outliers (%)	1.1
**Ramachandran**	
Favoured (%)	96.0
Disallowed (%)	0.1

Supplementary Table 2 – Single particle cryoEM statistics
