# Supplementary material for: CryoEM reveals that ribosomes in microsporidian spores are locked in a dimeric hibernating state
Source: Nat Microbiol. 2023 Sep 14;8(10):1834–45. doi: 10.1038/s41564-023-01469-w (PMC10522483; doi:10.1038/s41564-023-01469-w)
Supplement: Supplementary file 2 — Reporting Summary [file 41564_2023_1469_MOESM2_ESM.pdf]

nature portfolio

Corresponding author(s): Bertram Daum

Last updated by author(s): Jul 24, 2023

Reporting Summary

Nature Portfolio wishes to improve the reproducibility of the work that we publish. This form provides structure for consistency and transparency in reporting. For further information on Nature Portfolio policies, see our Editorial Policies and the Editorial Policy Checklist.

Statistics

For all statistical analyses, confirm that the following items are present in the figure legend, table legend, main text, or Methods section.

n/a	Confirmed	
☐	☒	The exact sample size (*n*) for each experimental group/condition, given as a discrete number and unit of measurement
☐	☒	A statement on whether measurements were taken from distinct samples or whether the same sample was measured repeatedly
☒	☐	The statistical test(s) used AND whether they are one- or two-sided<br>*Only common tests should be described solely by name; describe more complex techniques in the Methods section.*
☒	☐	A description of all covariates tested
☒	☐	A description of any assumptions or corrections, such as tests of normality and adjustment for multiple comparisons
☐	☒	A full description of the statistical parameters including central tendency (e.g. means) or other basic estimates (e.g. regression coefficient) AND variation (e.g. standard deviation) or associated estimates of uncertainty (e.g. confidence intervals)
☒	☐	For null hypothesis testing, the test statistic (e.g. *F*, *t*, *r*) with confidence intervals, effect sizes, degrees of freedom and *P* value noted<br>*Give P values as exact values whenever suitable.*
☒	☐	For Bayesian analysis, information on the choice of priors and Markov chain Monte Carlo settings
☒	☐	For hierarchical and complex designs, identification of the appropriate level for tests and full reporting of outcomes
☒	☐	Estimates of effect sizes (e.g. Cohen's *d*, Pearson's *r*), indicating how they were calculated

Our web collection on statistics for biologists contains articles on many of the points above.

Software and code

Policy information about availability of computer code

Data collection	SerialEM v3.7, EPU v2.8.1 (Thermo Fisher Scientific), Xcalibur2.1 (Thermoscientific)
Data analysis	Aretomo v1.2.5, IMOD v4.12.16, Relion v3.1, Warp v1.0.9, DeepEMhancer (Sep 2020), Coot v0.9, Chimera v1.15, CCP4 v8.0, Molrep v11.9, Alphafold2, Isolde v1.0, ChimeraX v1.3, CCPEM v1.4, Proteome Discoverer v2.1 (Thermo Scientific)

For manuscripts utilizing custom algorithms or software that are central to the research but not yet described in published literature, software must be made available to editors and reviewers. We strongly encourage code deposition in a community repository (e.g. GitHub). See the Nature Portfolio guidelines for submitting code & software for further information.

Data

Policy information about availability of data

All manuscripts must include a data availability statement. This statement should provide the following information, where applicable:

- Accession codes, unique identifiers, or web links for publicly available datasets
- A description of any restrictions on data availability
- For clinical datasets or third party data, please ensure that the statement adheres to our policy

The atomic coordinates of the S. lophii ribosome monomer and the single particle reconstruction density maps were deposited to the Protein Data Bank (https://www.rcsb.org) with accession number 7QCA and to the Electron Microscopy Data Bank (https://www.ebi.ac.uk/emdb) with the accession number EMD-13892. The sub-tomogram average of the S. lophii ribosome dimer and the coordinates of two ribosome particles fitted into it were deposited to the Protein Data Bank with

accession number 8P60 and EMD-17457. The sub-tomogram average of one half of the S. lophii ribosome dimer and the coordinates of one ribosome particle fitted into it were deposited to the Protein Data Bank with accession number 8P5D and EMD-17448. The mass spectrometry data has been submitted to MicrosporidiaDB (microsporidiadb.org). The previously determined structures of ribosomes of V. necatrix (6RM3), S. cerevisiae (5T62, 3J77, 3J78), S. scrofa (3J7P), E. coli (6H4N), E. cuniculi (7QEP), and P. locustae (6ZU5) used in this study are available from the PDB under the accession codes listed.

Human research participants

Policy information about studies involving human research participants and Sex and Gender in Research.

Reporting on sex and gender	No human research participants
Population characteristics	No human research participants
Recruitment	No human research participants
Ethics oversight	No human research participants

Note that full information on the approval of the study protocol must also be provided in the manuscript.

Field-specific reporting

Please select the one below that is the best fit for your research. If you are not sure, read the appropriate sections before making your selection.

☒ Life sciences ☐ Behavioural & social sciences ☐ Ecological, evolutionary & environmental sciences

For a reference copy of the document with all sections, see nature.com/documents/nr-reporting-summary-flat.pdf

Life sciences study design

All studies must disclose on these points even when the disclosure is negative.

Sample size	No specific statistical methods were used to determine sample size as it was not generally applicable to our study. For electron microscopy data, sample size was determined by the availability of areas to image on grids. Sample sizes are sufficiently large to be statistically relevant (n = 27,510 images and 977,879 particles for single particle dataset; n = 6505 particles for tomography dataset).
Data exclusions	No data were excluded.
Replication	Ribosome sample preps were replicated 6 times and spore germination was replicated 10 times.
Randomization	Randomization was not relevant as particles for structure determination were picked automatically.
Blinding	Blinding was not relevant as no data were excluded and possible biased evaluation steps were calculated using established software.

Reporting for specific materials, systems and methods

We require information from authors about some types of materials, experimental systems and methods used in many studies. Here, indicate whether each material, system or method listed is relevant to your study. If you are not sure if a list item applies to your research, read the appropriate section before selecting a response.

Materials & experimental systems

n/a	Involved in the study	
☒	☐	Antibodies
☒	☐	Eukaryotic cell lines
☒	☐	Palaeontology and archaeology
☐	☒	Animals and other organisms
☒	☐	Clinical data
☒	☐	Dual use research of concern

Methods

n/a	Involved in the study	
☒	☐	ChIP-seq
☒	☐	Flow cytometry
☒	☐	MRI-based neuroimaging

Animals and other research organisms

Policy information about studies involving animals; ARRIVE guidelines recommended for reporting animal research, and Sex and Gender in Research

Laboratory animals	n/a
Wild animals	Parasites were isolated from wild fish tissue acquired commercially, for example donated as non consumable fish tissue from fishmongers or trawler agents or fish were purchased whole and fish tissue was removed for consumption before infected tissue was taken to the lab for parasite isolation.
Reporting on sex	*Indicate if findings apply to only one sex; describe whether sex was considered in study design, methods used for assigning sex. Provide data disaggregated for sex where this information has been collected in the source data as appropriate; provide overall numbers in this Reporting Summary. Please state if this information has not been collected. Report sex-based analyses where performed, justify reasons for lack of sex-based analysis.*
Field-collected samples	n/a
Ethics oversight	As fish were obtained commercially from fishmongers, this type of research on fish tissue does not require ethical review by the University

Note that full information on the approval of the study protocol must also be provided in the manuscript.
